# Systemic kappa opioid receptor antagonism accelerates reinforcement learning via augmentation of novelty processing in male mice

**DOI:** 10.1038/s41386-023-01547-x

**Published:** 2023-02-17

**Authors:** Zahra Z. Farahbakhsh, Keaton Song, Hannah E. Branthwaite, Kirsty R. Erickson, Snigdha Mukerjee, Suzanne O. Nolan, Cody A. Siciliano

**Affiliations:** Department of Pharmacology, Vanderbilt Brain Institute, Vanderbilt Center for Addiction Research, Vanderbilt University, Nashville, TN 37232 USA

**Keywords:** Motivation, Operant learning

## Abstract

Selective inhibition of kappa opioid receptors (KORs) is highly anticipated as a pharmacotherapeutic intervention for substance use disorders and depression. The accepted explanation for KOR antagonist-induced amelioration of aberrant behaviors posits that KORs globally function as a negative valence system; antagonism thereby blunts the behavioral influence of negative internal states such as anhedonia and negative affect. While effects of systemic KOR manipulations have been widely reproduced, explicit evaluation of negative valence as an explanatory construct is lacking. Here, we tested a series of falsifiable hypotheses generated a priori based on the negative valence model by pairing reinforcement learning tasks with systemic pharmacological KOR blockade in male C57BL/6J mice. The negative valence model failed to predict multiple experimental outcomes: KOR blockade accelerated contingency learning during both positive and negative reinforcement without altering innate responses to appetitive or aversive stimuli. We next proposed novelty processing, which influences learning independent of valence, as an alternative explanatory construct. Hypotheses based on novelty processing predicted subsequent observations: KOR blockade increased exploration of a novel, but not habituated, environment and augmented the reinforcing efficacy of novel visual stimuli in a sensory reinforcement task. Together, these results revise and extend long-standing theories of KOR system function.

## Introduction

Central and peripheral kappa opioid receptors (KORs) are targets of a wide variety of pharmacotherapeutics. Most currently approved compounds are multi-target drugs, with varying pharmacodynamic actions and selectivity for KORs. These drugs are used for the treatment of a diverse array of disorders including, but not limited to, nalfurafine for pruritus [1], alfentanil for nociceptive [2] and levorphanol for neuropathic pain [3], naltrexone for substance use disorders [4], and eluxadoline for irritable bowel syndrome [5, 6]. Development of selective KOR compounds has been a longstanding goal in medicinal chemistry and recent breakthroughs have produced clinically viable, highly selective KOR agonists and antagonists, several of which are in various stages of clinical evaluation (National Clinical Trial number: NCT02800928, NCT02218736, NCT01913535, NCT02641028, NCT02475447) [1, 7, 8]. In particular, clinically viable selective KOR antagonists have been highly anticipated due to wide consensus in the preclinical literature that systemic blockade of KORs holds great promise for the treatment of several neuropsychiatric disorders including depression, substance use disorders, and anxiety [9–12].

In preclinical models, KOR activity has been causally linked to the underlying behavioral symptomatology of multiple neuropsychiatric diseases. For example, systemic administration of KOR antagonists in model species reliably reverses escalated drug and alcohol consumption resulting from chronic exposure [13–17], ameliorates depression-like phenotypes [18, 19], and can prevent the behavioral consequences of chronic stress [20–23]. It is thought that KOR modulation of addiction-, depression-, and anxiety-related behaviors stems from its endogenous function as a negative valence system. Indeed, the prevailing and widely accepted model of KOR’s role in neuropsychiatric disease posits that acute activation of KORs produces dysphoria and that experience-dependent upregulation of this system drives aberrant behavioral states such as anhedonia in depression and negative affect during periods of drug abstinence in addiction [24–41]. This model of the KOR’s primary role in neurobehavioral processes centers on negative valence processing and motivated behaviors driven by aversive internal states, which are both critical but latent constructs (i.e., cannot be directly observed). Though the latent nature of the variables involved does not allow for a straightforward operationalized definition, the theory has been semantically formalized by multiple literatures. The theory states that KOR activity is involved in negative valence domains (e.g., acute threat, potential threat, sustained threat, loss, and frustrative nonreward) and is critical for the development of subsequent behavioral responses to aversive stimuli, such as negative reinforcement [12, 22, 25, 42, 43]. The role of KORs in these constructs are typically tested in behavioral assays such as learned helplessness [44], forced swim stress [18], stress-induced reinstatement [45, 46], and intracranial self-stimulation [47].

Despite robust and widely reproduced findings that KORs are directly involved in symptomologies associated with substance use and mood disorders [13, 15, 17, 18, 48, 49], few studies have systematically evaluated a priori predictions from the negative valence model of KOR function, which represents a critical missing link in determining the veracity and utility of this framework [50–53]. Thus, we sought to directly evaluate predictions of the negative valence model of KOR function in mice during reinforcement learning – a quantitative framework recognized across disciplines for its utility in investigating fundamental processes relevant to basic functions and disease states [54–57]. We found that the negative valence model was insufficient to explain valence-independent experimental outcomes of KOR antagonism and thus proposed novelty processing – a construct which is critical for the identification and learning of stimuli with reinforcement-predictive value – as an *a posteriori* explanation. Generating axiomatic hypotheses based on novelty processing as a latent construct underlying KOR modulation of behavior predicted multiple experimental outcomes whereby systemic KOR antagonism augmented measures of novelty exploration and novelty-driven intrinsic motivation. Together, these findings call for re-evaluation of long-standing theories regarding KOR system function and delineate KOR control of a conserved neurobehavioral domain broadly implicated in motivated behaviors.

## Methods and materials

### Subjects

Adult, male C57BL/6J mice were used for all experiments (Jackson Laboratory, Bar Harbor, ME). Mice were group-housed (5 per cage) in a reverse 12-hour light-dark cycle room. Water was available *ad libitum* and 2.8–3 g of chow per mouse (Labdiet 5L0D) was provided daily, sufficient to maintain mice at healthy adult weights throughout the course of experiments (20–30 g bodyweight). All experiments involving the use of mice were in accordance with NIH guidelines and approved by the Vanderbilt University Institutional Animal Care and Use Committee.

### Drugs

The selective and potent KOR antagonist norbinaltorphimine (NorBNI) [58] was graciously provided by the NIDA Drug Supply Program. Mice were treated with NorBNI (10 mg/kg) or saline in a volume of 10 mL/kg intraperitoneally (i.p.) 24 h prior to the start of behavioral testing. NorBNI is long-acting, and extensive pharmacodynamic and pharmacokinetic analyses show that it remains detectable in the central nervous system for at least 21 days following a single injection, concomitant with inactivation of KORs over this time period [59–61].

### Positive reinforcement

During positive reinforcement experiments, operant boxes were equipped with a nose-poke port on either side, a small cue light above each port, a liquid delivery port in the center, and a house light on the opposite wall. One nose-poke port was active (counterbalanced across mice) on which responses in the presence of a discriminative stimulus (S^D^) resulted in the delivery of sucrose. The other nose-poke inactive with no consequent stimulus to a response. On a variable-time schedule ranging from 20–40 s (average 30 s), a cue light above the active side was illuminated for up to 30 s and served as an S^D^. A response on the active side during the S^D^ period was deemed a ‘correct response’ and resulted in the delivery of 10 µL of a 10% sucrose solution (w/v), the illumination of a cue light above the liquid delivery port, and the termination of the S^D^. During the interval between S^D^ presentations, referred to as the S^Δ^ period (i.e., a condition negatively correlated with reinforcement) [62–67], a response on the active side resulted in a 30 s timeout period signaled by the illumination of a house light. Responses on either port during the timeout period had no consequence and the variable-time scheduled was discontinued such that no other stimuli were presented. After 30 s had elapsed, the house light terminated, signaling the beginning of another S^Δ^ period and resumption of variable-time schedule.

### Negative reinforcement

During negative reinforcement experiments, the operant chamber and general procedures were the same as described above, but behavior was reinforced by electrification of the metal grid floor (footshock). Counterbalanced between mice, one nose-poke port was an active port on which responses in the presence of the S^D^ resulted in the avoidance or escape from footshocks, and the other was inactive with no consequent stimulus to a response. On a variable-time schedule ranging from 20–40 s (average 30 s), a cue light above the active side was illuminated (S^D^). If no response was made within 30 s of S^D^ onset, a series of 20 mild (0.15 mA) footshocks would begin. Within the series of footshocks, each footshock was 0.5 s in duration and there was a period of 15 s between the offset of one footshock and the onset of the next. The S^D^ remained illuminated during the series of footshocks and was extinguished after all 20 shocks were delivered or after a correct response was made. A response on the active side during the first 30 s of the S^D^ resulted in the complete avoidance of footshocks and a 1-minute extension of the S^Δ^ period. A response on the active side after the footshock series began would result in the escape from the remainder of the series concomitant with beginning of the next S^Δ^ period. Both responses on the active side which resulted in the avoidance and escape of the footshocks were deemed ‘correct responses.’ Unlike in the positive reinforcement task (where a response during the S^Δ^ period resulted in a timeout), during negative reinforcement a response on the active side during the S^Δ^ period had no consequence. Consistent with the positive reinforcement task, any response on the inactive side had no consequence.

### Crossover treatment

After meeting acquisition criteria (positive reinforcement, see supplemental methods) or after the 15th session (negative reinforcement), a subset of mice received the opposite treatment that they were given at the beginning of reinforcement learning at least 30 min after removal from the operant box. Thus, saline pretreated mice were given NorBNI post-acquisition (saline→NorBNI) and vice versa (NorBNI→saline). Subjects were tested over three sessions following the crossover treatment under identical experimental conditions as described above. For each mouse that received the crossover treatment, post-treatment performance was calculated by normalizing to pretreatment values ([average 3 days post]/[average 3 days pre]x100).

### Novelty response

At least 24 h after i.p. treatment with NorBNI (10 mg/kg) or saline, mice performed the first (Day 1, novel) of two open field tests. On day 1, each mouse was placed in the center of the arena and allowed to explore for the duration of the 1 h session, after which they were immediately removed from the apparatus. The following day (Day 2, familiar), mice were re-tested using the same procedure. Distance traveled (cm) per 5 minute bin throughout both sessions was calculated with Noldus Ethovision video-tracking.

### Sensory reinforcement

#### Fixed-ratio 1

A seperate cohort of mice was trained on a fixed-ratio 1 (FR 1) task in a box equipped with one response lever (side counterbalanced) and three stimulus lights in the center for 5 1-hour sessions during which a response on the lever would result in randomized light flashes and a response during the reinforcer period had no consequence. Flash duration (4, 6, 8, and 10 s) and frequency (0.5, 1, 2.5, 5, 10, 12.5, and 25 Hz) were randomized for each reinforcer earned. For each flash of light, the cue light (top, middle, bottom) which was illuminated was also randomized. At least 30 min after the fifth session, mice were treated with saline or NorBNI (10 mg/kg). The next day, mice ran the same FR 1 task to determine if treatment altered responding for the novel stimuli.

#### Behavioral economics

The behavioral economics procedure was designed such that mice had 10 min to respond for the novel stimuli at each price. For the first 10 min of the session the price was 1 lever press (FR 1) and the mouse was only constrained by the number of reinforcers that would fit in the time window. During the following 10 min the price was 3 lever presses (FR 3) for a reinforcer and so on with ratios FR 5, 10, 20, 30, and 60. A demand curve was fit using the equation $\log Q = \log Q_0 + k\left( {e^{ - a \times \left( {Q_0 \times C} \right)} - 1} \right)$ to derive Q_0_, standardized P_max_, and O_max_, as previously described [68]. Q_0_ is the consumption as price approaches zero, standardized P_max_ is the first unit price point at which the first derivative point slope of the function equals −1 multiplied by Q_0_ to standardize, and O_max_ is the number of responses at P_max_.

## Results

### Systemic KOR antagonism accelerated discriminated operant responding during positive reinforcement learning

**Fig. 1:**
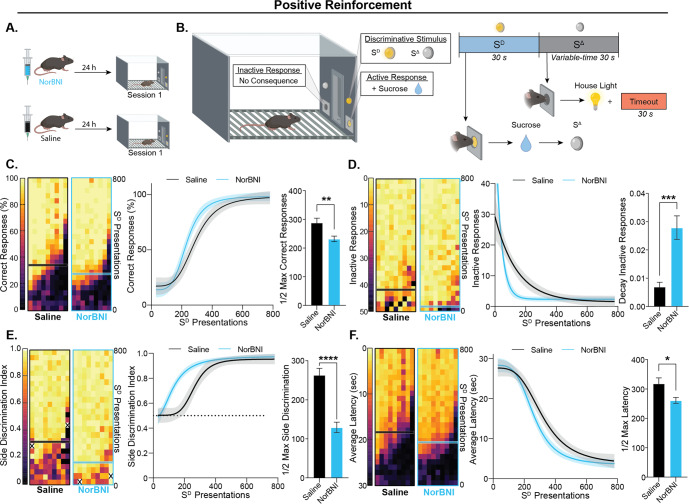
Systemic KOR antagonism increased rate of positive reinforcement learning. **A** Schematic of experimental design. Mice were i.p. injected with NorBNI or saline 24 h prior to the first behavioral session. **B** Schematic of the positive reinforcement task structure. A response on the active side in the presence of the S^D^ (i.e., correct response) resulted in the delivery of a sucrose solution to a liquid delivery port. A response on the active side in the absence of the S^D^ (S^Δ^ period) results in a 30-second timeout indicated by the illumination of the house light and a response on the inactive side has no consequence. **C**–**F** *Left:* Heatmaps show each individual subject’s performance across 800 S^D^ presentations with half-max performance determined from the group data indicated by a horizontal line. *Center:* Data were fit with to describe the learning curve across time/experience. Best nonlinear curve fit by group is shown with a 95% confidence band. *Right:* The best fit half-max or decay constant between groups was compared using an unpaired t-test. **C** Mice treated with NorBNI reached half-maximal performance on the probability of emitting a correct response ([correct responses/S^D^ presentations]x100) in fewer S^D^ presentations than saline treated controls (unpaired t-test, *t*_16_ = 3.063, *p* = 0.0074). **D** The NorBNI group had a faster rate of decay in inactive responding compared to the saline control group (unpaired t-test, *t*_16_ = 4.707, *p* = 0.0002). **E** Mice that received NorBNI learned to discriminate between the active and inactive sides more quickly, taking fewer S^D^ presentations to reach half-maximal side discrimination index (active side responses/all responses) compared to saline controls (unpaired t-test, *t*_16_ = 6.222, *p* < 0.0001). An ‘X’ in the heatmap indicates that no response was made during the S^D^ presentation bin, and thus a side discrimination index could not be calculated. **F** Mice treated with NorBNI reached half-maximal latency to respond following S^D^ onset in fewer presentations than saline-treated controls (unpaired t-test, *t*_16_ = 2.676, *p* = 0.0166). Values indicate mean ± SEM unless otherwise noted. (*n* = 9 per group) (*p ≤ 0.05, **p ≤ 0.01, ***p ≤ 0.001, ****p ≤ 0.0001).

Given the prevalence of valence processing frameworks for interpreting KOR control of motivated behaviors, we first sought to examine how modulation of this system altered learning reinforced by positive and negative operant contingencies where differentially valenced stimuli can be evaluated under analogous experimental conditions [69]. Based on literature positing that KOR activation mediates responses to aversive stimuli and thus drives negative reinforcement, we hypothesized that KOR antagonism would selectively impair negative reinforcement learning with minimal impact on positive reinforcement. To test this hypothesis, mice were given a single i.p. injection of NorBNI (10 mg/kg) or saline 24 h prior to the first behavioral session (Fig. 1A).

Positive reinforcement learning was tested in an operant box in daily, one-hour sessions (Fig. 1B). In contrast to our hypothesis, we found that a single injection of NorBNI increased the rate of discriminated positive reinforcement learning across multiple measures of performance (Fig. 1C–F). Analysis of temporal patterns of responding over sessions revealed that NorBNI treatment decreased the number of S^D^ presentations necessary to reach half-maximal probability of emitting a correct response during the stimulus presentation (Fig. 1C), indicative of augmented acquisition of the discriminated operant contingency. This was not due to increased responding in general as the number of responses on the inactive port decreased more rapidly in mice treated with NorBNI compared to saline controls (Fig. 1D). Congruently, NorBNI treatment robustly accelerated discrimination between the active and inactive nose-poke ports as assessed by a side discrimination index (Fig. 1E). KOR antagonism also increased the rate at which mice learned to optimize response latency following S^D^ onset (Fig. 1F). This surprising effect of KOR antagonism on learning rate under a positive reinforcement contingency was not due to weight differences between groups nor a difference in the ability of mice to eventually acquire the task (Supplementary Fig. 1A, B). Together, these findings suggest that NorBNI augments learning even when behavior is reinforced under a positive contingency by a reinforcer with positive valence.

### KOR antagonism selectively increased learning rate without impacting maximal performance

**Fig. 2:**
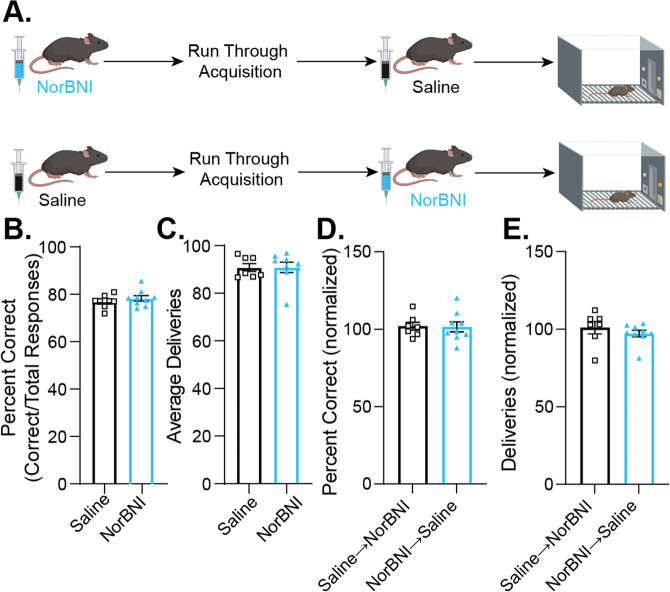
Systemic KOR blockade did not alter performance of a previously learned contingency during positive reinforcement. Acquisition criteria was set to 4 out of 5 consecutive days with greater than 70% of responses being correct and at least 40 reinforcers earned. **A** Schematic of experimental design for crossover treatment. After acquisition, a subset of mice received a crossover treatment in which mice that had previously received a saline injection received an injection of NorBNI (10 mg/kg), and vice versa, at least 30 min after the operant session and roughly 20 h prior to the next session. **B**, **C** There is no effect of NorBNI on the average performance over the 4 sessions during which acquisition criteria were met, which served as the baseline measurement for crossover comparison. **B** Over the 4 days at or above acquisition criteria, there is no difference between NorBNI and control mice in the percent correct by session ([correct responses/total responses] × 100) (unpaired t-test, *t*_14_ = 0.8028, *p* = 0.4355). **C** At acquisition, there is also no difference in the average number of sucrose deliveries between groups (unpaired t-test, *t*_14_ = 0.03204, *p* = 0.9749). **D**, **E** Comparison of performance following crossover treatment. Values represent average performance over the 3 days following the crossover treatment normalized to the average of the 3 days prior. **D** After subjects reached acquisition criteria, administration of NorBNI had no effect on percent correct responses compared to saline controls (unpaired t-test, *t*_14_ = 0.09605, *p* = 0.9248). **E** Treatment with NorBNI after learning also had no effect on the normalized number of sucrose deliveries per session compared to saline controls (unpaired t-test, *t*_14_ = 0.8911, *p* = 0.3879). Values indicate mean ± SEM. (*n* = 9, NorBNI→saline; *n* = 7, saline→NorBNI).

Despite its influence on learning rate, KOR blockade showed no effect on maximal performance across all the metrics examined (Supplementary Fig. 1C–F), suggesting that the impact of KOR blockade is specific to the acquisition of learned behaviors, as opposed to behavioral expression of previously learned knowledge. To directly evaluate this hypothesis, a subset of subjects was tested using a crossover design (Fig. 2A). Over the 4 days at or above acquisition criteria, there was no difference between groups in percent correct or average deliveries, confirming that prior to the crossover treatment both groups plateaued at the same level of task performance (Fig. 2B, C). NorBNI administered after task acquisition had no effect on percent correct responses or reinforcers earned per session, demonstrating that KOR blockade does not impact performance of the task after learning has occurred (Supplementary Fig. 2A, B, Fig. 2D, E). Thus, systemic antagonism of KORs accelerates positive reinforcement learning but has no effect on maximal performance or on a previously learned contingency.

### KOR modulation of learning rate was not due to altered consummatory response

From the results thus far, it is not clear whether NorBNI acts to modulate global variables affecting learning or, alternatively, alters the motivational value or preference for sucrose to produce a reinforcer-specific effect. To evaluate this possibility, we next analyzed sucrose consumption across positive reinforcement sessions. There were no differences between groups in average bout duration, lick bouts per sucrose delivery, or licks per bout at any point throughout the task (Supplementary Fig. 3A–F). This suggests that NorBNI had no effect on the motoric action of licking or consummatory response for the 10% sucrose solution used as a reinforcer during operant learning.

To test the impact of KOR blockade on fluid consumption and sucrose preference independent of reinforcement learning, we next conducted an open access two-bottle choice experiment. Within-subject, pre-post comparison revealed that NorBNI had no effect on overall licking or microstructure patterns for 1% sucrose (Supplementary Fig. 4A–C). NorBNI had no effect on consummatory behavior across the dose-response curve (Supplementary Fig. 4D–F). There was also no difference in the number of licks or the lick microstructure for water after treatment with either saline or NorBNI (Supplementary Fig. 5A–C). Together, these data show that KOR antagonism does not alter innate responses to sucrose, a stimulus with positive valence, and indicate that augmented rate of positive reinforcement learning is more likely to be driven by modulation of global constructs underlying acquisition of learned behaviors.

### Systemic KOR antagonism increased reinforcement learning rate independent of reinforcer valence

**Fig. 3:**
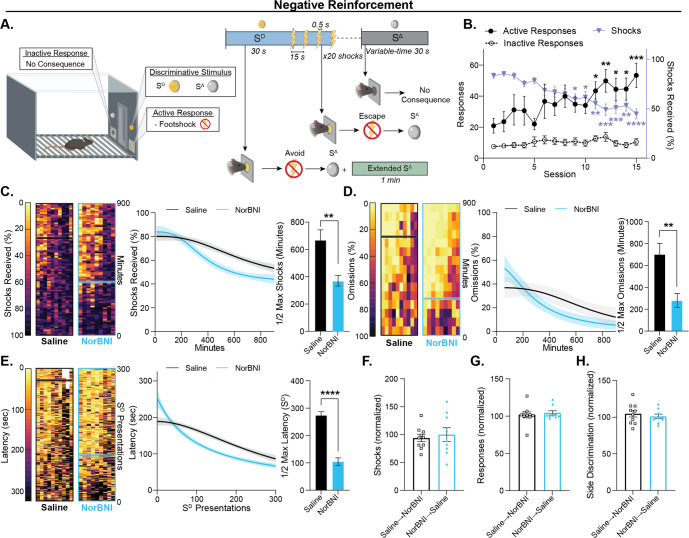
Systemic KOR blockade increased the rate of negative reinforcement learning. Mice were treated with NorBNI (10 mg/kg) or saline 24 h prior to their first session of a negative reinforcement task that paralleled the positive reinforcement task to determine whether the learning effect occurs across reinforcement contingencies. **A** Schematic of the negative reinforcement task. Failure to respond within 30 s of the S^D^ onset resulted in the initiation of a series of 20 mild (0.15 mA) footshocks. A correct response resulted in the avoidance (if response occurred before shock onset) or escape (termination of ongoing shocks) of the footshocks. A response on the active side during the S^Δ^ period and a response on the inactive side had no consequence. **B** Aggregate data demonstrating acquisition of the negative reinforcement contingency in mice. Mice increased the number of active side responses over sessions (repeated measures one-way ANOVA, sessions, *F*_(3.885, 69.92)_ = 2.775, *p* = 0.0349; Šidák multiple comparisons test to session 1 baseline). There was no change in the number of inactive side responses over sessions (repeated measures one-way ANOVA, sessions, *F*_(4.666, 83.98)_ = 1.134, *p* = 0.3482). Subjects showed a minimization in the proportion of possible shocks received over sessions (shocks received/theoretical maximum [220 per session]) (repeated measures one-way ANOVA, sessions, *F*_(4.439, 79.91)_ = 13.70, *p* < 0.0001; Šidák multiple comparisons test to session 1). **C**–**E** Measures of reinforcement learning binned by time or S^D^ presentations. *Left:* Heatmaps show each individual subject’s performance across learning with the best fit half-max values indicated by a horizontal line. *Center:* The best nonlinear curve fit by group is shown with a 95% confidence band over sessions. *Right:* The best fit half-maximal performance between groups was compared using a t-test. NorBNI increased the rate of negative reinforcement learning across measures of performance. **C** NorBNI mice took less time to reach half-maximal performance on percent of possible shocks received per 10 min (received/36 possible) (unpaired t-test, *t*_17_ = 3.484, *p* = 0.0028). **D** Mice that received NorBNI took less time to reach their half-maximal performance in the percent of omissions (S^D^ presentations with no response/total S^D^ presentations) (unpaired t-test, *t*_17_ = 3.491, *p* = 0.0028). **E** KOR blockade increased the rate at which mice optimized response latency (unpaired t-test, *t*_17_ = 8.603, *p* < 0.0001). **F**–**H** After 15 sessions, mice received a crossover treatment to test whether NorBNI would have an effect on performance after initial learning. At least 30 min after the operant session and 20 h prior to the next session, mice that initially received saline were given an i.p. injection of NorBNI (10 mg/kg) and vice versa. Mice continued reinforcement sessions for three days and changes in performance [(average performance 3 days preceding/average performance 3 days after) x 100] were measured. **F** Treatment with NorBNI after initial learning had no effect on the percent of shocks received (shocks/220 possible) compared to saline controls (unpaired t-test, *t*_17_ = 0.4703, *p* = 0.6441). **G** After initial learning, NorBNI had no effect on the percent of shock series avoided or escaped (correct responses/S^D^ presentations) compared to saline controls (unpaired t-test, *t*_17_ = 0.5281, *p* = 0.6043). **H** Likewise, there was no effect of the crossover treatment on side discrimination (active response/all responses) between groups (unpaired t-test, *t*_17_ = 0.6544, *p* = 0.5216). Values indicate mean ± SEM unless otherwise noted. (NorBNI, *n* = 9; saline, *n* = 10) (*p ≤ 0.05, **p ≤ 0.01, ***p ≤ 0.001, ****p ≤ 0.0001).

We next sought to directly evaluate the central prediction of the negative valence model: that KOR blockade disrupts processing of aversive stimuli thereby attenuating motivated behaviors driven by negative reinforcement contingencies. Mice were treated with either NorBNI or saline prior to the first negative reinforcement session (Fig. 3A). Over sessions, subjects increased responding on the active side without changing responding on the inactive side and received fewer shocks, demonstrating that footshocks functioned as a negative reinforcer under these conditions (Fig. 3B). In contrast to canonical theories of KOR function, antagonism increased the rate of negative reinforcement learning across measures of performance, mirroring KOR modulation of positive reinforcement learning (Fig. 3C–E). KOR blockade accelerated the decay rate over session time for shocks delivered (Fig. 3C), probability of response omission during the S^D^ period (Fig. 3D), and latency to respond following S^D^ onset (Fig. 3E). Similar to effects on positive reinforcement learning, KOR antagonism did not impact performance during negative reinforcement when treatment was given after initial learning had occurred (Supplemental Fig. 6A–C, Fig. 3F–H).

### KOR antagonism did not alter unconditioned responses to footshock

To investigate whether NorBNI treatment alters the unconditioned response to footshock, mice without any prior experimental experience received non-contingent, unsignaled footshocks in an operant chamber. Analysis of locomotion aligned to footshock onset revealed a time-locked, intensity-dependent increase in velocity in response to footshock, as expected (Supplementary Fig. 7A). However, NorBNI treatment did not alter responsiveness to footshock over any of the intensities tested (Supplementary Fig. 7B, C). This demonstrates that KOR blockade does not alter unconditioned responses to footshock, which is incongruent with a central tenant of the negative valence model and furthers supports the hypothesis that KOR modulation of learning rate is independent of reinforcer valence and modality.

### KOR antagonism selectively augmented novelty exploration independent of habituation rate, general ambulatory activity, and neophobic avoidance behaviors

Though the results are incongruent with the negative valence model, the experiments above do not provide an alternative explanation for KOR control of learning. Thus, we next aimed to identify a construct congruent with our results which could be used to generate falsifiable axiomatic hypotheses. Human, animal, and in silico studies have reached broad consensus that novelty processing is a critical construct which influences reinforcement learning independent of stimulus valence and primary motivational drives maintaining response-reinforcer associations [70–74]. This factor is particularly relevant for acquisition of stimulus-response-reinforcer contingencies where behavior comes under the control of a neutral, antecedent stimulus due to its function as an S^D^ rather than as a primary reinforcer [75–77]. Increased novelty processing augments learning rate by allocating increased attention towards, and/or assigning intrinsic value to, novel choices and stimuli, which has pronounced effects on learning to associate neutral cues with availability of primary reinforcers without impacting learning curve asymptote [70, 78–80]. In sum, the impact of increased novelty exploration on learning is congruent with the effects of KOR blockade observed in the experiments above. Therefore, if novelty exploration is modulated by KOR antagonism it may provide a more holistic explanation for its impact on motivated behaviors.

**Fig. 4:**
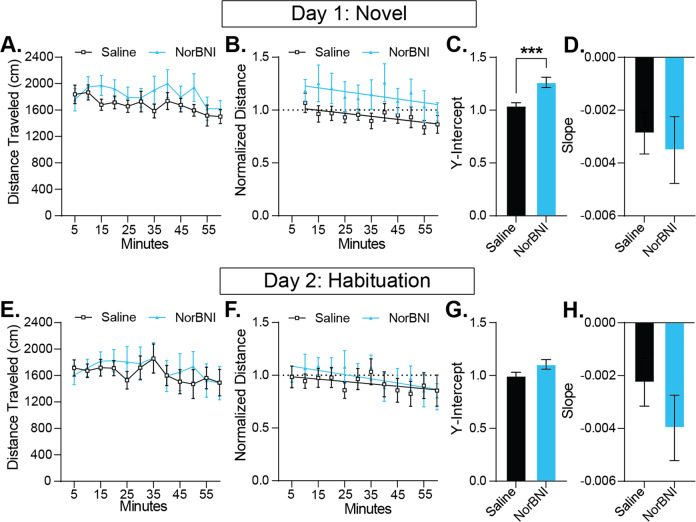
KOR antagonism increased novelty exploration. **A** Raw distance traveled binned by 5 min during the first hour-long session in a novel open field. **B** Distance traveled was normalized within-subject to the distance traveled in the first 5 min of exposure to the open field. The relative exploratory behavior was fit with a linear curve (saline, df = 9, r^2^ = 0.5942; NorBNI, df = 9, r^2^ = 0.4615). **C** NorBNI increased relative exploratory behavior over the first session in the open field as the Y-intercept was higher than that of saline controls (unpaired t-test, *t*_22_ = 3.881, *p* = 0.0008). **D** Though NorBNI increased the amount of exploratory behavior in response to novelty, KOR antagonism did not change the rate of habituation as the slope was not different between groups (unpaired t-test, *t*_22_ = 0.4295, *p* = 0.6717). **E** The distance traveled during the second hour-long session in the open field, once it is more familiar, was binned by 5 min. **F** The distance traveled during the second session was binned and normalized to the first 5 min of exposure to the open field during the novel session with the best linear fit (saline, df = 10, r^2^ = 0.3867; NorBNI, df = 10, r^2^ = 0.5077). **G** Mice that received NorBNI had a trending increase in relative exploratory behavior during the second session (unpaired t-test, *t*_22_ = 1.929, *p* = 0.0667). **H** There was no difference in the rate of habituation during the second session between subjects (unpaired t-test, *t*_22_ = 1.124, *p* = 0.2731). Values indicate mean ± SEM. (*n* = 12 per group) (**p ≤ 0.01).

To test the hypothesis that systemic KOR blockade augments responsiveness to novelty, we used a well-validated measure of novelty exploration: locomotor response to a novel environment and habituation of exploratory behavior over experience [81–84]. Absolute distance traveled per five minutes followed a predictable time-course whereby activity was pronounced early in the session, eventually reaching a peak after which movement steadily decreased over the remainder of the 60 min (Fig. 4A). Normalizing activity to the first five minutes of the session to account for variance unrelated to exploratory drive allows clear assessment of the novelty-driven exploration phase, eventually followed by a steady decrease in locomotion over time dictated by habituation rate (Fig. 4B). Systemic KOR blockade by NorBNI augmented the degree to which activity increased above baseline but did not alter the rate at which locomotion decreased over the session (Fig. 4C, D). This resulted in a considerably prolonged duration of novel environment-induced increases in activity whereby NorBNI treated mice did not return to baseline activity until the end of the 60-minute session as opposed to 15 min or less in controls.

In contrast to when the environment was novel, there was no group differences in the degree to which activity was increased above baseline initially or the rate at which locomotor activity habituated over time during the second session when the environment was familiar (Fig. 4E–H), confirming that differences observed in the first session are specific to a novel environment, and that KOR blockade does not appear to influence spontaneous locomotor activity. Further, increased novelty-induced exploratory behavior was not associated with reciprocal changes in neophobic processes, as additional analysis revealed that novelty-dependent avoidance of the center of the open field area did not differ between groups during either session (Supplemental Fig. 8A–D).

### KOR blockade augmented essential reinforcing value of novel stimuli

**Fig. 5:**
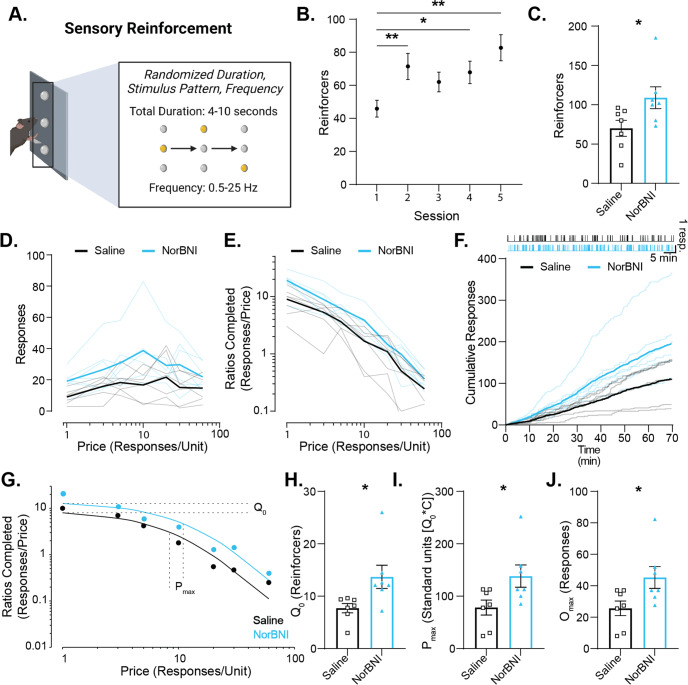
Systemic KOR blockade increased the intrinsic motivational value of novel stimuli. **A** Schematic of the sensory reinforcement task whereby an active response resulted in a randomized pattern of light illumination. **B** Novel sensory stimuli were reinforcing, as responding increased across sessions (repeated measures one-way ANOVA, sessions, *F*_(3.35, 43.54)_ = 9.065, *p* < 0.0001; Šidák multiple comparisons test to session 1 baseline). **C** After training, subjects were treated with NorBNI or saline and underwent an additional FR 1 session. NorBNI increased the number of sensory stimuli reinforcers earned on a FR 1 schedule (unpaired t-test, *t*_12_ = 2.259, *p* = 0.0433). **D**–**J** The following day, mice underwent a behavioral economics session during which the price (responses/unit) increased across discrete 10 min time bins. Responses per time bin were recorded and curve fit to extract measures of intrinsic value and motivation for novel stimuli. **D** The number of responses made at each price, with light/thin lines indicating individual subjects and dark lines indicating group averages. **E** The number of ratios completed (responses/price) during each time bin with individual values indicated in light colored lines and NorBNI and saline averages indicated by dark lines. **F** Cumulative records of active responses made during the behavioral economics session with individual records indicated with light lines and group averages indicated with dark lines. Representative response records from one saline-treated and one NorBNI-treated subject an are along the top of the graph with each upward tick indicating one response made. **G** Representative demand curves from an individual subject from each group with P_max_ indicated. **H** KOR blockade increased reinforcers earned at a minimally constraining price (Q_0_) (unpaired t-test, *t*_12_ = 2.510, *p* = 0.0274). **I** NorBNI increased the motivation for novel stimuli as measured by the standardized P_max_ (Q_0_*C) (unpaired t-test, *t*_12_ = 2.346, *p* = 0.0370). **J** The number of responses made at P_max_, or O_max_, was higher in mice that were treated with NorBNI compared to saline (unpaired t-test, *t*_12_ = 2.349, *p* = 0.0368). Values indicate mean ± SEM. (*n* = 7 per group) (*p ≤ 0.05, **p ≤ 0.01).

To directly assess novelty-driven reinforcement without habituation of responding, we utilized a sensory reinforcement task where responding is reinforced by identical visual stimuli throughout but longitudinal behavior can be maintained by randomizing the pattern and frequency of each stimulus presentation (Fig. 5A, Supplementary Video 1) [85–87]. Randomized illumination of the cue lights presented on a FR 1 schedule maintained robust responding which increased over sessions, demonstrating that the stimulus reliably functioned as a reinforcer under these conditions and is insensitive to habituation (Fig. 5B).

Treatment with NorBNI increased the number of novel reinforcers earned under a FR 1 schedule (Fig. 5C). Though responding under an FR 1 contingency demonstrates that novel sensory stimuli are more reinforcing with KOR blockade, the minimal effort required to make one response makes continuous reinforcement insufficient to measure motivation for presentation of the stimulus. To better assess novelty-driven intrinsic motivation, we used a within-session threshold procedure to quantify demand elasticity, or the degree to which consumption of a commodity changes as a function of price – a widely accepted measurement of essential motivational properties of reinforcers [88, 89]. The number of responses made at each price, the number of ratios completed (consumption), and the response records were recorded (Fig. 5D-F). A demand curve was fit to extract behavioral economic parameters for each subject **(**Fig. 5G**)**. Q_0_ is the subject’s preferred level of commodity consumption, or the amount the subject would consume at a minimally constraining price. Standardized P_max_ represents the amount consumed at the price at which demand becomes elastic and the subject stops responding sufficiently to maintain the desired level of consumption, and O_max_ is the number of responses made at P_max_. Mice treated with NorBNI showed more motivation for novel stimuli, with an increase in Q_0_, standardized P_max_, and O_max_ (Fig. 5H–J).

To ensure that augmented motivation was specific to responding for novel stimuli rather than any sensory stimuli or responding per se, a separate cohort was tested using a continuous, 30 s cue light illumination as a reinforcer which did not vary across presentations (Supplemental Fig. 9A). There was no difference in responding for this stimulus between saline and NorBNI mice (Supplemental Fig. 9B) suggesting that the effect of NorBNI on intrinsic motivation is specifically novelty-driven. Together, these experiments demonstrate that KOR blockade increases the intrinsic motivational value of novel stimuli and provides further evidence supporting the utility of novelty processing as an explanatory construct underlying KOR modulation of motivated behaviors.

## Discussion

Here, we assessed predictions of the negative valence model of KOR function starting with the hypothesis that systemic inhibition of KOR activity would not alter positive reinforcement but would decrease the rate of behaviors reinforced under a negative contingency. We measured the effect of KOR antagonism on positive and negative reinforcement learning and found that, contrary to our hypothesis, KOR blockade accelerated the rate of learning both under a positive and negative contingency, regardless of reinforcer valence. Augmented performance was selective to learning/task acquisition as previously learned behaviors and maximal performance were unaffected. Further, KOR antagonism did not alter innate responses to sucrose or footshock, as would be expected if valence processing were modulated. Together, our results demonstrate that a negative valence framework for the KOR system does not accurately predict effects of KOR modulation on basic behaviors.

Importantly, while our findings are incongruent with standing theories, they are not in conflict with the empirical results in the literature. Studies demonstrating beneficial effects of KOR antagonists that have been interpreted within the negative valence framework have typically examined single endpoints (e.g., reduced drug and alcohol intake under free-access conditions, reduced immobility during forced swim test). Drawing causal inferences with any latent construct necessitates intersectional analysis of multiple observable variables and, as a corollary, any observable effect taken in isolation can be explained by several underlying latent constructs [90–92]. As such, the latent constructs responsible for the pharmacotherapeutic actions of KOR antagonists remains debatable. Though the putative therapeutic effects of KOR antagonism have been widely reproduced within specific model paradigms, they often do not generalize across experimental setting and disparate conclusions can be found throughout the literature with studies demonstrating no effect of KOR antagonism [93, 94], evidence of KOR agonists both increasing and decreasing substance use [95–103], and results showing anxiolytic effects of KOR agonism [104, 105]. Furthermore, there are many instances of investigations linking KOR activation in specific circuits and brain regions with functions outside of negative valence processing. For example, intra-striatal microinjections of KOR agonists can increase hedonic response to sucrose, induce conditioned place preference, and decrease anxiety-like behaviors depending on the subregion targeted [106, 107]. Similarly, optogenetic activation of subsets of dynorphin releasing neurons can drive opposing effects on place preference behavior depending on anatomical location within the ventral striatum [108]. Intriguingly, our results are highly congruent with recent clinical investigations demonstrating that KOR antagonism in mood disorder patients augments behavioral flexibility and learning rate without affecting hedonic responses to primary rewards [109, 110].

Although multiple lines of evidence have demonstrated complexity in the role for the KOR system beyond mediating only aversion and dysphoria, as has long been accepted, previous findings have typically been interpreted as circuit-specific effects of KOR activation rather than as incongruent with the global theory of the system. Having directly evaluated the negative valence model, we next sought to identify a construct that could explain our results and potentially provide better predictive validity moving forward. We found that systemic KOR blockade augments one of the strongest innate drivers of behavior – novelty processing. The influence of KORs on novelty processing has potentially wide-reaching implications; indeed, attraction to the unknown is a prerequisite for higher-order knowledge and is thought to influence virtually all aspects of human and animal behavior [111–114], including learning [78, 115, 116], behavioral flexibility [117–119], and even pain processing [120–124]. Regarding reinforcement learning specifically, the ability of an organism to recognize and respond to novelty is critical to its ability to adapt to the environment and the tendency for novelty to stimulate exploratory behaviors guarantees diverse experiences required for learning complex contingencies [85, 125, 126]. As such, novelty processing plays a key role in operant reinforcement task acquisition and highly influences the rate of learning through a variety of mechanisms [78, 127]. For example, due to the stochasticity of initial interactions with the operandum, as they have no known value to the subject, increased exploration of a novel environment can augment learning simply by increasing the probability of triggering the operant contingency. More importantly, heightened intrinsic reinforcing value associated with novelty or increased allocation of attention towards novel stimuli can increase the salience and the likelihood of long-term encoding of initial action-outcome pairings [128, 129]. Thus, increased response to novel environments and stimuli augments learning rate by modulating attention, motivation, and memory formation during operant tasks [127].

Future work exploring how KOR regulation of novelty processing modulates additional forms of learning and behavioral flexibility through reversal, extinction, or set-shifting tasks (for example) will be critical to understanding the complex way in which this system is implicated in behavior. Though the effect of KOR blockade on novelty processing suggests a role for endogenous signaling in this process, it is worth noting that this does not exclude the possibility that exogenous activation of the system modulates other behavioral processes. It is also important to consider that this work, and that which served as the foundation of the negative valence model, were conducted with male mice and there is a wide range of literature suggesting major sex differences in effects of KOR activity [130–135], thus, further work is certainly required to understand the role of the KOR system in motivated behaviors in females. Despite these limitations, evidence that systemic KOR antagonism modulates novelty processing provides a new avenue for understanding the role of this system in a variety of adaptive and maladaptive behaviors.

As mentioned above, widely reproduced effects of KOR antagonists in preclinical substance use disorder and depression models can be putatively explained by several underlying constructs, including modulation of novelty processing. For example, decreased responding during drug self-administration can also fit a novelty exploration model, as augmented novelty exploration is likely to reduce rates of ongoing behavior by assigning value to novel choices [136]. Likewise, increased exploration of a novel environment may slow the emergence of immobility during forced swim assays [137]. The complex interplay between anxiety and novelty whereby an increase in novelty processing could present either as anxiolytic (neophilia) or anxiogenic (neophobia) behavior depending on the magnitude of novelty and the intensity of the stimulus [77] also suggests that this model may fit the wealth of literature implicating the KOR system in anxiety-like behaviors [138–140]. Assessing these possibilities goes well beyond the scope of the current report, and we do not claim that the previous results demonstrate causality of altered novelty exploration, nor that claims of negative valence modulation are necessarily false. It is certainly conceivable that negative valence processing explains the effects of KOR antagonism under some specific conditions. Rather, our results directly challenge the utility of the negative valence framework as the primary model of KOR function and call for re-evaluation, via empirical assessment, of conclusions that draw from this framework.

## Supplementary information

Supplemental Video Legend

Supplement

Supplemental Video
