# Supplementary material for: Systemic kappa opioid receptor antagonism accelerates reinforcement learning via augmentation of novelty processing in male mice
Source: Neuropsychopharmacology. 2023 Feb 17;48(6):857–68. doi: 10.1038/s41386-023-01547-x (PMC10156709; doi:10.1038/s41386-023-01547-x)
Supplement: Supplementary file 1 — Supplemental Video Legend [file 41386_2023_1547_MOESM1_ESM.docx]

**Supplemental Video 1.** Overhead video of an animal performing the sensory reinforcement task. Under a fixed-ratio 1 schedule, behavior was reinforced by illumination of three identical cue lights for a random duration, at a random frequency, and in a random pattern.
