# Supplementary material for: Systemic kappa opioid receptor antagonism accelerates reinforcement learning via augmentation of novelty processing in male mice
Source: Neuropsychopharmacology. 2023 Feb 17;48(6):857–68. doi: 10.1038/s41386-023-01547-x (PMC10156709; doi:10.1038/s41386-023-01547-x)
Supplement: Supplementary file 2 — Supplement [file 41386_2023_1547_MOESM2_ESM.docx]

Supplementary Materials for

**Systemic kappa opioid receptor antagonism accelerates reinforcement learning via augmentation of novelty processing**

Zahra Z. Farahbakhsh, Keaton Song, Hannah E. Branthwaite, Kirsty R. Erickson, Snigdha Mukerjee, Suzanne O. Nolan, Cody A. Siciliano*

Department of Pharmacology, Vanderbilt Brain Institute, Vanderbilt Center for Addiction Research, Vanderbilt University, Nashville, TN 37232, USA.

*Corresponding Author Email: cody.siciliano@vanderbilt.edu

**Supplemental Table 1. Statistics associated with main figures.**

| Associated Figure  Dependent Measure | Groups (n) | Test (design) | Test Statistic | | p value | Summary |
| --- | --- | --- | --- | --- | --- | --- |
| **Figure 1C** | Saline (9) | t test (unpaired) | t | 3.063 | 0.0074 | ** |
| Correct Responses (%) | NorBNI (9) |  | df | 16 |  |  |
|  |  |  |  |  |  |  |
| **Figure 1D** | Saline (9) | t test (unpaired) | t | 4.707 | 0.0002 | *** |
| Inactive Responses | NorBNI (9) |  | df | 16 |  |  |
| **Figure 1E**  Side Discrimination | Saline (9)  NorBNI (9) | t test (unpaired) | t  df | 6.222  16 | < 0.0001 | **** |
| Index |  |  |  |  |  |  |
| **Figure 1F** | Saline (9) | t test (unpaired) | t | 2.676 | 0.0166 | * |
| Average Latency (s) | NorBNI (9) |  | df | 16 |  |  |
|  |  |  |  |  |  |  |
| **Figure 2B** | Saline (7) | t test (unpaired) | t | 0.8028 | 0.4355 | ns |
| Percent Correct | NorBNI (9) |  | df | 14 |  |  |
|  |  |  |  |  |  |  |
| **Figure 2C** | Saline (7) | t test (unpaired) | t | 0.03204 | 0.9749 | ns |
| Average Deliveries | NorBNI (9) |  | df | 14 |  |  |
|  |  |  |  |  |  |  |
| **Figure 2D** | NorBNI🡪Saline (9) | t test (unpaired) | t | 0.09605 | 0.9248 | ns |
| Percent Correct (normalized) | Saline🡪NorBNI (7) |  | df | 14 |  |  |
|  |  |  |  |  |  |  |
| **Figure 2E** | NorBNI🡪Saline (9) | t test (unpaired) | t | 0.8911 | 0.3879 | ns |
| Deliveries (normalized) | Saline🡪NorBNI (7) |  | df | 14 |  |  |
|  |  |  |  |  |  |  |
| **Figure 3B**  Active Responses | Subjects (19) | One-way ANOVA (repeated measures) | F DFn | 2.775  3.885 | 0.0349 | * |
|  |  |  | DFd | 69.92 |  |  |
|  |  | Šidák multiple comparisons test |  |  |  |  |
|  |  | Day 1 vs 2  Day 1 vs 3 |  |  | >0.9999  0.9851 | ns  ns |
|  |  | Day 1 vs 4  Day 1 vs 5 |  |  | 0.9977  >0.9999 | ns  ns |
|  |  | Day 1 vs 6  Day 1 vs 7 |  |  | 0.9357  0.7607 | ns  ns |
|  |  | Day 1 vs 8  Day 1 vs 9 |  |  | 0.2637  0.1792 | ns  ns |
|  |  | Day 1 vs 10  Day 1 vs 11 |  |  | 0.3575  0.0177 | ns  * |
|  |  | Day 1 vs 12  Day 1 vs 13 |  |  | 0.0074  0.0465 | **  * |
|  |  | Day 1 vs 14  Day 1 vs 15 |  |  | 0.0226  0.0004 | *  *** |
| **Figure 3B**  Inactive Responses | Subjects (19) | One-way ANOVA (repeated measures) | F DFn | 1.134  4.666 | 0.3482 | ns |
|  |  |  | DFd | 83.98 |  |  |
|  |  | Šidák multiple comparisons test |  |  |  |  |
|  |  | Day 1 vs 2  Day 1 vs 3 |  |  | >0.9999  >0.9999 | ns  ns |
|  |  | Day 1 vs 4  Day 1 vs 5 |  |  | >0.9999  0.9986 | ns  ns |
|  |  | Day 1 vs 6  Day 1 vs 7 |  |  | 0.9155  0.9741 | ns  ns |
|  |  | Day 1 vs 8  Day 1 vs 9 |  |  | 0.9946  0.9880 | ns  ns |
|  |  | Day 1 vs 10  Day 1 vs 11 |  |  | 0.9987  0.1633 | ns  ns |
|  |  | Day 1 vs 12  Day 1 vs 13 |  |  | 0.4740  0.9977 | ns  ns |
|  |  | Day 1 vs 14  Day 1 vs 15 |  |  | >0.9999  0.7689 | ns  ns |
| **Figure 3B**  Shocks Received | Subjects (19) | One-way ANOVA (repeated measures) | F DFn | 13.70  4.439 | < 0.0001 | **** |
| (%) |  |  | DFd | 79.91 |  |  |
|  |  | Šidák multiple comparisons test |  |  |  |  |
|  |  | Day 1 vs 2  Day 1 vs 3 |  |  | >0.9999  >0.9999 | ns  ns |
|  |  | Day 1 vs 4  Day 1 vs 5 |  |  | 0.4710  0.9701 | ns  ns |
|  |  | Day 1 vs 6  Day 1 vs 7 |  |  | 0.1839  0.1837 | ns  ns |
|  |  | Day 1 vs 8  Day 1 vs 9 |  |  | 0.0826  0.0150 | ns  * |
|  |  | Day 1 vs 10  Day 1 vs 11 |  |  | 0.0487  0.0019 | *  ** |
|  |  | Day 1 vs 12  Day 1 vs 13 |  |  | 0.0002  0.0004 | ***  *** |
|  |  | Day 1 vs 14  Day 1 vs 15 |  |  | 0.0021  <0.0001 | **  **** |
| **Figure 3C** | Saline (10) | t test (unpaired) | t | 3.484 | 0.0028 | ** |
| Shocks Received (%) | NorBNI (9) |  | df | 17 |  |  |
|  |  |  |  |  |  |  |
| **Figure 3D** | Saline (10) | t test (unpaired) | t | 3.491 | 0.0028 | ** |
| Omissions (%) | NorBNI (9) |  | df | 17 |  |  |
| **Figure 3E**  Latency (s) | Saline (10)  NorBNI (9) | t test (unpaired) | t  df | 8.603  17 | < 0.0001 | **** |
| **Figure 3F**  Shocks (normalized) | Saline (10)  NorBNI (9) | t test (unpaired) | t  df | 0.4703  17 | 0.6441 | ns |
| **Figure 3G**  Responses (normalized) | Saline (10)  NorBNI (9) | t test (unpaired) | t  df | 0.5281  17 | 0.6043 | ns |
| **Figure 3H**  Side Discrimination (normalized) | Saline (10)  NorBNI (9) | t test (unpaired) | t  df | 0.6544  17 | 0.5216 | ns |
| **Figure 4C** | Saline (12) | t test (unpaired) | t | 3.881 | 0.0008 | *** |
| Novel Y-Intercept | NorBNI (12) |  | df | 22 |  |  |
| **Figure 4D** | Saline (12) | t test (unpaired) | t | 0.4295 | 0.6717 | ns |
| Novel Slope | NorBNI (12) |  | df | 22 |  |  |
| **Figure 4G** | Saline (12) | t test (unpaired) | t | 1.929 | 0.0667 | ns |
| Habituation Y-Intercept | NorBNI (12) |  | df | 22 |  |  |
| **Figure 4H** | Saline (12) | t test (unpaired) | t | 1.124 | 0.2731 | ns |
| Habituation Slope | NorBNI (12) |  | df | 22 |  |  |
| **Figure 5B**  Reinforcers | Subjects (14) | One-way ANOVA (repeated measures) | F DFn | 9.065  3.35 | <0.0001 | **** |
|  |  |  | DFd | 43.54 |  |  |
|  |  | Šidák multiple comparisons test |  |  |  |  |
|  |  | Day 1 vs 2  Day 1 vs 3 |  |  | 0.0037  0.1148 | **  ns |
|  |  | Day 1 vs 4  Day 1 vs 5 |  |  | 0.0238  0.0020 | *  ** |
| **Figure 5C** | Saline (7) | t test (unpaired) | t | 2.259 | 0.0433 | * |
| Reinforcers | NorBNI (7) |  | df | 12 |  |  |
| **Figure 5H** | Saline (7) | t test (unpaired) | t | 2.510 | 0.0274 | * |
| Q_0_ | NorBNI (7) |  | df | 12 |  |  |
| **Figure 5I** | Saline (7) | t test (unpaired) | t | 2.346 | 0.0370 | * |
| P_max_ (Standard Units) | NorBNI (7) |  | df | 12 |  |  |
| **Figure 5J** | Saline (7) | t test (unpaired) | t | 2.349 | 0.0368 | * |
| O_max_ (Responses) | NorBNI (7) |  | df | 12 |  |  |

**Supplemental Table 2. Statistics associated with supplemental figures.**

| Associated Figure  Dependent Measure | Groups (n) | Test (design) | Test Statistic | | p value | Summary |
| --- | --- | --- | --- | --- | --- | --- |
| **Supp. Figure 1A** | Saline (9) | t test (unpaired) | t | 0.228 | 0.8225 | ns |
| Average Weight (g) | NorBNI (9) |  | df | 16 |  |  |
| **Supp. Figure 1B** | Saline (9) | Chi-squared | χ^2^ | 0.0 | > 0.9999 | ns |
| Proportion Acquired | NorBNI (9) |  |  |  |  |  |
| **Supp. Figure 1C** | Saline (9) | t test (unpaired) | t | 0.001785 | 0.9986 | ns |
| Max. Correct Responses (%) | NorBNI (9) |  | df | 16 |  |  |
| **Supp. Figure 1D** | Saline (9) | t test (unpaired) | t | 0.7898 | 0.4412 | ns |
| Min. Inactive Responses | NorBNI (9) |  | df | 16 |  |  |
|  |  |  |  |  |  |  |
| **Supp. Figure 1E** | Saline (9) | t test (unpaired) | t | 0.9083 | 0.3772 | ns |
| Max. Side Discrimination | NorBNI (9) |  | df | 16 |  |  |
|  |  |  |  |  |  |  |
| **Supp. Figure 1F** | Saline (9) | t test (unpaired) | t | 0.03784 | 0.9703 | ns |
| Max. Latency (s) | NorBNI (9) |  | df | 16 |  |  |
| **Supp. Figure 3A** | Saline (9) | two-way mixed-model |  |  |  |  |
| Bout Duration (s) | NorBNI (9) | ANOVA |  |  |  |  |
|  |  | Session | F | 4.869 | 0.0022 | ** |
|  |  |  | DFn | 3.809 |  |  |
|  |  |  | DFd | 57.56 |  |  |
|  |  | Treatment | F | 0.2213 | 0.6444 | ns |
|  |  |  | DFn | 1 |  |  |
|  |  |  | DFd | 16 |  |  |
|  |  | Session x Treatment | F | 0.5977 | 0.9002 | ns |
|  |  |  | DFn | 18 |  |  |
|  |  |  | DFd | 272 |  |  |
| **Supp. Figure 3B** | Saline (9) | two-way mixed-model |  |  |  |  |
| Licks per Delivery | NorBNI (9) | ANOVA |  |  |  |  |
| (s) |  | Session | F | 7.553 | 0.0014 | ** |
|  |  |  | DFn | 2.239 |  |  |
|  |  |  | DFd | 33.58 |  |  |
|  |  | Treatment | F | 0.3597 | 0.5571 | ns |
|  |  |  | DFn | 1 |  |  |
|  |  |  | DFd | 16 |  |  |
|  |  | Session x Treatment | F | 0.5117 | 0.9518 | ns |
|  |  |  | DFn | 18 |  |  |
|  |  |  | DFd | 270 |  |  |
| **Supp. Figure 3C** | Saline (9) | two-way mixed-model |  |  |  |  |
| Bouts per Delivery | NorBNI (9) | ANOVA |  |  |  |  |
|  |  | Session | F | 7.530 | 0.0016 | ** |
|  |  |  | DFn | 2.188 |  |  |
|  |  |  | DFd | 32.70 |  |  |
|  |  | Treatment | F | 0.2913 | 0.5968 | ns |
|  |  |  | DFn | 1 |  |  |
|  |  |  | DFd | 16 |  |  |
|  |  | Session x Treatment | F | 0.5837 | 0.9101 | ns |
|  |  |  | DFn | 18 |  |  |
|  |  |  | DFd | 269 |  |  |
| **Supp. Figure 3D** | Saline (9) | t test (unpaired) |  |  |  |  |
| Bout Duration (s) | NorBNI (9) | Early | t | 0.7111 | 0.4873 | ns |
|  |  |  | df | 16 |  |  |
|  |  | Middle | t | 0.6949 | 0.4971 | ns |
|  |  |  | df | 16 |  |  |
|  |  | Late | t | 0.02974 | 0.9766 | ns |
|  |  |  | df | 16 |  |  |
| **Supp. Figure 3E** | Saline (9) | t test (unpaired) |  |  |  |  |
| Licks per Delivery | NorBNI (9) | Early | t | 0.9395 | 0.3614 | ns |
|  |  |  | df | 16 |  |  |
|  |  | Middle | t | 0.2327 | 0.8189 | ns |
|  |  |  | df | 16 |  |  |
|  |  | Late | t | 0.7314 | 0.4751 | ns |
|  |  |  | df | 16 |  |  |
| **Supp. Figure 3F** | Saline (9) | t test (unpaired) |  |  |  |  |
| Bouts per Delivery | NorBNI (9) | Early | t | 0.7806 | 0.4464 | ns |
|  |  |  | df | 16 |  |  |
|  |  | Middle | t | 0.3518 | 0.7296 | ns |
|  |  |  | df | 16 |  |  |
|  |  | Late | t | 0.6360 | 0.5337 | ns |
|  |  |  | df | 16 |  |  |
| **Supp. Figure 4A** | Saline (7) | two-way mixed-model |  |  |  |  |
| Licks | NorBNI (8) | ANOVA |  |  |  |  |
|  |  | Session | F | 0.3566 | 0.5606 | ns |
|  |  |  | DFn | 1 |  |  |
|  |  |  | DFd | 13 |  |  |
|  |  | Treatment | F | 0.1560 | 0.6993 | ns |
|  |  |  | DFn | 1 |  |  |
|  |  |  | DFd | 13 |  |  |
|  |  | Šidák multiple comparisons test |  |  |  |  |
|  |  | Saline Pre- vs Post- | t  df | 0.06970  13 | 0.9970 | ns |
|  |  | NorBNI Pre- vs Post- | t | 0.7997 | 0.6844 | ns |
|  |  |  | df | 13 |  |  |
| **Supp. Figure 4B** | Saline (7) | two-way mixed-model |  |  |  |  |
| Bout Number | NorBNI (8) | ANOVA |  |  |  |  |
|  |  | Session | F | 0.8555 | 0.3719 | ns |
|  |  |  | DFn | 1 |  |  |
|  |  |  | DFd | 13 |  |  |
|  |  | Treatment | F | 0.0004467 | 0.9835 | ns |
|  |  |  | DFn | 1 |  |  |
|  |  |  | DFd | 13 |  |  |
|  |  | Šidák multiple comparisons test |  |  |  |  |
|  |  | Saline Pre- vs Post- | t  df | 0.4115  13 | 0.9023 | ns |
|  |  | NorBNI Pre- vs Post- | t | 0.9141 | 0.6123 | ns |
|  |  |  | df | 13 |  |  |
| **Supp. Figure 4C** | Saline (7) | two-way mixed-model |  |  |  |  |
| Licks per Bout | NorBNI (8) | ANOVA |  |  |  |  |
|  |  | Session | F | 2.152 | 0.1662 | ns |
|  |  |  | DFn | 1 |  |  |
|  |  |  | DFd | 13 |  |  |
|  |  | Treatment | F | 0.004255 | 0.9490 | ns |
|  |  |  | DFn | 1 |  |  |
|  |  |  | DFd | 13 |  |  |
|  |  | Šidák multiple comparisons test |  |  |  |  |
|  |  | Saline Pre- vs Post- | t  df | 0.5629  13 | 0.8262 | ns |
|  |  | NorBNI Pre- vs Post- | t | 1.546 | 0.2710 | ns |
|  |  |  | df | 13 |  |  |
| **Supp. Figure 4D** | Saline (7) | t test (unpaired) |  |  |  |  |
| Licks | NorBNI (8) | Half-Max | t | 0.4787 | 0.6401 | ns |
|  |  |  | df | 13 |  |  |
|  |  | Curve Max | t | 0.3366 | 0.7418 | ns |
|  |  |  | df | 13 |  |  |
| **Supp. Figure 4E** | Saline (7) | t test (unpaired) |  |  |  |  |
| Bout Number | NorBNI (8) | Half-Max | t | 0.4251 | 0.6777 | ns |
|  |  |  | df | 13 |  |  |
|  |  | Curve Max | t | 0.5169 | 0.6139 | ns |
|  |  |  | df | 13 |  |  |
| **Supp. Figure 4F** | Saline (7) | t test (unpaired) |  |  |  |  |
| Average Licks per Bout | NorBNI (8) | Half-Max | t | 0.3482 | 0.7333 | ns |
|  |  |  | df | 13 |  |  |
|  |  | Curve Max | t | 0.7281 | 0.4794 | ns |
|  |  |  | df | 13 |  |  |
| **Supp. Figure 5A** | Saline (7) | two-way mixed-model |  |  |  |  |
| Licks | NorBNI (8) | ANOVA |  |  |  |  |
|  |  | Session | F | 1.092 | 0.3151 | ns |
|  |  |  | DFn | 1 |  |  |
|  |  |  | DFd | 13 |  |  |
|  |  | Treatment | F | 0.1358 | 0.7184 | ns |
|  |  |  | DFn | 1 |  |  |
|  |  |  | DFd | 13 |  |  |
|  |  | Šidák multiple comparisons test |  |  |  |  |
|  |  | Saline Pre- vs Post- | t  df | 0.8878  13 | 0.6289 | ns |
|  |  | NorBNI Pre- vs Post- | t | 0.5807 | 0.8163 | ns |
|  |  |  | df | 13 |  |  |
| **Supp. Figure 5B** | Saline (7) | two-way mixed-model |  |  |  |  |
| Bouts | NorBNI (8) | ANOVA |  |  |  |  |
|  |  | Session | F | 0.05743 | 0.8143 | ns |
|  |  |  | DFn | 1 |  |  |
|  |  |  | DFd | 13 |  |  |
|  |  | Treatment | F | 0.1249 | 0.7294 | ns |
|  |  |  | DFn | 1 |  |  |
|  |  |  | DFd | 13 |  |  |
|  |  | Šidák multiple comparisons test |  |  |  |  |
|  |  | Saline Pre- vs Post- | t | 0.08807 | 0.9953 | ns |
|  |  |  | df | 13 |  |  |
|  |  | NorBNI Pre- vs Post- | t | 0.2566 | 0.9606 | ns |
|  |  |  | df | 13 |  |  |
| **Supp. Figure 5C** | Saline (7) | two-way mixed-model |  |  |  |  |
| Licks per Bout | NorBNI (8) | ANOVA |  |  |  |  |
|  |  | Session | F | 1.198 | 0.2935 | ns |
|  |  |  | DFn | 1 |  |  |
|  |  |  | DFd | 13 |  |  |
|  |  | Treatment | F | 1.966 | 0.1843 | ns |
|  |  |  | DFn | 1 |  |  |
|  |  |  | DFd | 13 |  |  |
|  |  | Šidák multiple comparisons test |  |  |  |  |
|  |  | Saline Pre- vs Post- | t | 1.447 | 0.3137 | ns |
|  |  |  | df | 13 |  |  |
|  |  | NorBNI Pre- vs Post- | t | 0.05563 | 0.9981 | ns |
|  |  |  | df | 13 |  |  |
| **Supp. Figure 7B** | Saline (5) | t test (unpaired) |  |  |  |  |
| Peak | NorBNI (5) | Half-Max | t | 0.2022 | 0.8448 | ns |
|  |  |  | df | 8 |  |  |
|  |  | Curve Max | t | 0.07231 | 0.9441 | ns |
|  |  |  | df | 8 |  |  |
| **Supp. Figure 7C** | Saline (5) | t test (unpaired) |  |  |  |  |
| AUC | NorBNI (5) | Half-Max | t | 0.4442 | 0.6687 | ns |
|  |  |  | df | 8 |  |  |
|  |  | Curve Max | t | 1.088 | 0.3081 | ns |
|  |  |  | df | 8 |  |  |
| **Supp. Figure 8A** | Saline (12) | two-way ANOVA |  |  |  |  |
| Center Entries | NorBNI (12) | Time | F | 4.629 | 0.0003 | *** |
| Novel |  |  | DFn | 5.786 |  |  |
|  |  |  | DFd | 127.3 |  |  |
|  |  | Treatment | F | 0.005713 | 0.9404 | ns |
|  |  |  | DFn | 1 |  |  |
|  |  |  | DFd | 22 |  |  |
|  |  | Time x Treatment | F | 0.5805 | 0.8440 | ns |
|  |  |  | DFn | 11 |  |  |
|  |  |  | DFd | 242 |  |  |
| **Supp. Figure 8B** | Saline (12) | two-way ANOVA |  |  |  |  |
| Center Time Novel | NorBNI (12) | Time | F | 6.087 | < 0.0001 | **** |
|  |  |  | DFn | 6.354 |  |  |
|  |  |  | DFd | 139.8 |  |  |
|  |  | Treatment | F | 0.9989 | 0.3284 | ns |
|  |  |  | DFn | 1 |  |  |
|  |  |  | DFd | 22 |  |  |
|  |  | Time x Treatment | F | 1.460 | 0.1473 | ns |
|  |  |  | DFn | 11 |  |  |
|  |  |  | DFd | 242 |  |  |
| **Supp. Figure 8C** | Saline (12) | two-way ANOVA |  |  |  |  |
| Center Entries | NorBNI (12) | Time | F | 5.971 | < 0.0001 | **** |
| Habituation |  |  | DFn | 4.848 |  |  |
|  |  |  | DFd | 106.7 |  |  |
|  |  | Treatment | F | 1.071 | 0.3121 | ns |
|  |  |  | DFn | 1 |  |  |
|  |  |  | DFd | 22 |  |  |
|  |  | Time x Treatment | F | 0.7502 | 0.6894 | ns |
|  |  |  | DFn | 11 |  |  |
|  |  |  | DFd | 242 |  |  |
| **Supp. Figure 8D** | Saline (12) | two-way ANOVA |  |  |  |  |
| Center Time | NorBNI (12) | Time | F | 5.703 | 0.0001 | **** |
| Habituation |  |  | DFn | 4.762 |  |  |
|  |  |  | DFd | 104.8 |  |  |
|  |  | Treatment | F | 1.713 | 0.2041 | ns |
|  |  |  | DFn | 1 |  |  |
|  |  |  | DFd | 22 |  |  |
|  |  | Time x Treatment | F | 1.403 | 0.1721 | ns |
|  |  |  | DFn | 11 |  |  |
|  |  |  | DFd | 242 |  |  |
| **Supp. Figure 9B** | Saline (9) | two-way ANOVA |  |  |  |  |
| Reinforcers | NorBNI (10) | Time | F | 5.077 | 0.0073 | ** |
|  |  |  | DFn | 2.409 |  |  |
|  |  |  | DFd | 40.96 |  |  |
|  |  | Treatment | F | 1.147 | 0.2992 | ns |
|  |  |  | DFn | 1 |  |  |
|  |  |  | DFd | 17 |  |  |
|  |  | Time x Treatment | F | 0.2620 | 0.9013 | ns |
|  |  |  | DFn | 4 |  |  |
|  |  |  | DFd | 68 |  |  |

**Supplemental Materials and Methods**

**Acquisition and Exclusion Criteria**

*Positive Reinforcement*

Acquisition criteria was defined as over 70% correct (correct responses/total responses) and at least 40 sucrose deliveries over 4 out of 5 consecutive days (these criteria were set based on prior experiments in the lab demonstrating that performance asymptotes at this point). Two mice, one from each treatment group, did not reach acquisition criteria within 19 days/sessions and were excluded from further analysis. Data loss due to a computer crash resulted in a skipped day of recorded behavior for one cohort (n = 4, NorBNI; n = 5, saline) on what would have been the 14th day of behavior. Therefore, one cohort had a day of behavior excluded between sessions 13 and 14.

*Negative Reinforcement*

If mice did not avoid at least 25% of shocks per session after 15 sessions, they were excluded from analysis (one mouse was excluded based on this criteria).

**Two-Bottle Sucrose Preference Assay**

To measure differences in sucrose preference independent of learned responses, we conducted an open access two-bottle choice experiment with a separate cohort of mice. Each operant box was equipped with a bottle on either side, wired to a resistance contact lickometer, with one bottle containing a sucrose solution and the other containing water (side was counterbalanced between mice on session one and bottle location was switched for each consecutive session to counterbalance across days). The day prior to the first session, mice were primed with sucrose in the home cage so that the first exposure to sucrose would not be within the operant box. Mice first underwent lick training to remove the rate at which mice learned that sucrose was available as a confound of the response to sucrose. For lick training, mice were given open access to a bottle of 1% (w/v) sucrose and a bottle of water for as long as it took to reach 250 licks on the sucrose bottle. After lick training, mice underwent three days of 30-minute baseline sessions with open access to a bottle of 1% sucrose and a bottle of water. After the baseline sessions, mice were treated with NorBNI (10 mg/kg) or saline and, at least 24 hours later, ran a session with two-bottle choice access to 1% sucrose and water for a within-subject comparison. Then, the first cohort of mice (n = 3, NorBNI; n = 2, saline) was run on a dose response curve with access to one concentration a day (5%, 10%). To extend the width of the dose response curve after the within-subject comparison, the second cohort (n = 5 each group) ran on 0.5%, 1%, 5%, and 10%.

**Lick Microstructure**

Microstructure analysis of lick contacts during positive reinforcement sessions was conducted using custom MATLAB code. The following definitions were used in the analysis, based on previous literature^1,2^. We extracted the microstructural patterns of lick behavior, which is a well-validated analysis that allows detection of subtle differences in taste reactivity and consummatory behaviors based on frequency and size of individual lick bouts^3^. The lick duration was defined as the time between lick onset and offset. A bout was defined as 3 licks within 1 second of each other, with the onset of the bout being the first of the 3 licks. Bout termination was defined as when there was a 3 second pause in licking. Bout size was defined as the number of licks in a bout and was averaged across all the bouts in a single session for every mouse. Bout size and the number of licks were then normalized to the number of deliveries the mouse received to account for differences in positive reinforcement performance. If a mouse had no deliveries or licks in a session, they were excluded from that day (for microstructure analysis only).

For lick microstructure analysis of two-bottle choice, the same protocol was used as for the analysis of licks during positive reinforcement. The three days of baseline sucrose licks, bout number, and average licks per bout, were averaged within-subject and were compared to values from the 1% sucrose session directly following treatment with NorBNI or saline. Dose response curves were generated from the lick microstructure values across concentrations and the best sigmoidal curve fit half-maximal concentration and curve maximum response were compared between groups. Response to water was compared within subject before and after treatment with NorBNI or saline by comparing the average pretreatment values (average 3-day baseline) to the average water consumption patterns across all sessions after treatment.

**Shock Response**

To test the effect of NorBNI on the response to footshock, a separate cohort of mice received a series of unsignaled shocks in the operant box approximately 24 hours after receiving an i.p. injection of NorBNI (10 mg/kg) or saline. Each mouse was placed in the chamber and video was recorded with an overhead infrared camera (Security Camera Warehouse) recording at 30 frames per second. The house light and background white noise were on for the entirety of the session. Mice received 10 rounds of 10 footshocks of varying intensity in ascending order (0.01, 0.05, 0.10, 0.15, 0.20, 0.25, 0.30, 0.40, 0.60, 0.80 mA). Each footshock was 0.5 seconds long and there were 15 seconds between the offset of one shock and the onset of the next. Videos were then tracked using Noldus Ethovision center-point tracking and visually verified. Frame-by-frame velocity was then aligned to footshock onset which were timestamped in the video. These values were then downsampled to 15 frames per second by averaging across 2 frames to minimize outliers in velocity changes. Traces were then averaged within-subject for each intensity to account for any issues with video tracking during any round of shocks. The peak velocity was determined by calculating the average velocity during a rolling 0.5 second window and taking the maximum of those values per subject. The area under the curve of the 0.5 seconds prior to shock onset until 5 seconds after shock onset was calculated using MATLAB. Peak velocity and the area under the curve were plotted by intensity and curve fit with a hyperbolic function based on best fit. The best fit half-maximal shock intensity and curve maximum velocity response was then compared by group.

**Open Field Analysis**

The arena was also separated into center and surround areas, with the outer 75% of the perimeter of the open field being considered the ‘surround’ and the inner 25% square as the ‘center.’ Studies have demonstrated that the temporal patterns of, rather than the total, locomotor activity are critical to evaluating the response to novelty^4–6^. To evaluate the temporal patterns of movement, distance traveled was normalized within-subject to the first 5 minutes of exposure to the open field and was linearly fit based on the trend of habituation^7^. A linear regression was used because it provided a straightforward description of both the magnitude of increase (Y-intercept) as well as the rate of habituation (slope) without requiring an arbitrary split (e.g., early vs late epochs of the session). This allowed for the consideration of habituation as a more continuous process rather than artificially segmenting it (though data was binned to some degree to get measures of distance). As these C57BL/6J mice demonstrated a largely linear reduction in locomotion over time (especially when compared to Wistar rats which show an exponential decay) a linear curve was fit and parameters were extracted from the best fit. Conceptually, Y-intercept (elevation) was considered to be the relative amount of exploration and slope to be the rate of habituation.

Notably, even in rats, the large majority of novel environment response data has been analyzed using a within-group design whereby animals are separated into upper and lower thirds (tertiary splits) post-hoc based on the magnitude of novelty response (e.g., high responder/low responder model^8–10^). Thus, the variance of each group is highly limited compared to the population variance, and any influence of general locomotion on the outcome measures is made negligible by removing the middle third of the subjects. The analyses used here provide a method to quantify exploration for between-group designs in which all subjects are included.

**Continuous Illumination**

Another cohort was used to ensure that the effect was specific to novel sensory stimuli. A new cohort was used because of the long-lasting effect of NorBNI. Due to this, a within-subject design would require testing across at least 1-month, and responding would reflect a mixture of habituation, extinction, and pharmacokinetic clearance of NorBNI. For this task, the operant box was equipped with a nose-poke port on either side and 3 cue lights stacked upon one another in the center. On the first day of sensory reinforcement, a small piece of a sugary cereal was placed within each nose-poke port to encourage interaction with the operandum. Counterbalanced between mice, one side was active and the other inactive. A response on the inactive side had no consequence. A response on the active side resulted in the presentation of a single middle light, continuously for 30 seconds.

**Statistics**

Statistical analyses were performed using GraphPad Prism (GraphPad Software V9, Inc, La Jolla, CA) and MATLAB (Mathworks, Natick, MA). For all pairwise comparisons between two timepoints or groups, we utilized paired or unpaired two-tailed Student’s t-tests, respectively. Comparisons across three or more timepoints were made using one-way ANOVAs (followed by Tukey’s post-hoc test). For analyses of two or more categorical variables, two-way ANOVAs were performed with ordinary, repeated measures, or mixed-model designs where appropriate (followed by Šidák multiple comparisons post-hoc tests). Threshold for significance was placed at p < 0.05. All data are shown as mean ± standard error of the mean (SEM).


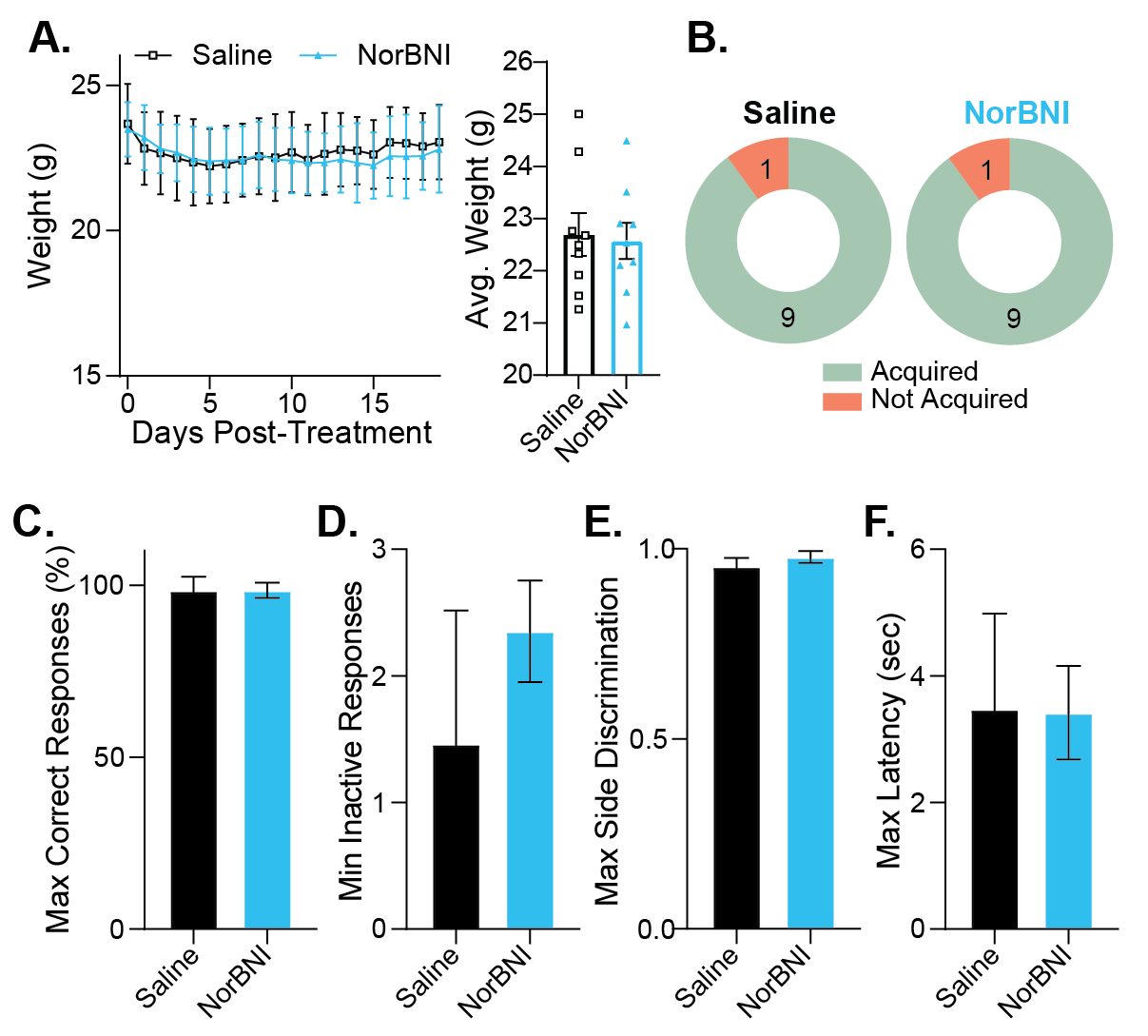


**Supplemental Figure 1. KOR antagonism did not affect maximal performance in positive reinforcement**. **(A)** Mouse weights between groups from the day of treatment through 19 days of positive reinforcement learning. NorBNI had no effect on the average weight of mice (unpaired t-test, t_16_ = 0.228, p = 0.8225). **(B)** Acquisition criteria for positive reinforcement learning was set to greater than 70% of responses occurring on the active side during the S^D^ (correct responses/total responses) and at least 40 sucrose deliveries for 4 out of 5 consecutive sessions. Each group (NorBNI and saline) had a 90% acquisition rate with 9 of 10 mice reaching criteria, suggesting no difference in the ability of groups to ultimately learn the task (Chi-squared, χ^2^ = 0.0, p > 0.9999). **(C-F)** Comparison of learning curve asymptote across measures of positive reinforcement acquisition. **(C)** NorBNI had no effect on the maximum percent of S^D^ presentations during which a correct response was made (unpaired t-test, t_16_ = 0.001785, p = 0.9986). **(D)** At asymptotic performance, there was no difference in the number of inactive responses between NorBNI and saline groups (unpaired t-test, t_16_ = 0.7898, p = 0.4412). **(E)** KOR blockade had no effect maximal side discrimination index acquired during positive reinforcement learning (unpaired t-test, t_16_ = 0.9083, p = 0.3772). **(F)** There was no difference in the maximal performance for response latency between groups (unpaired t-test, t_16_ = 0.03784, p = 0.9703). Values indicate mean ± SEM. (n = 9 per group)


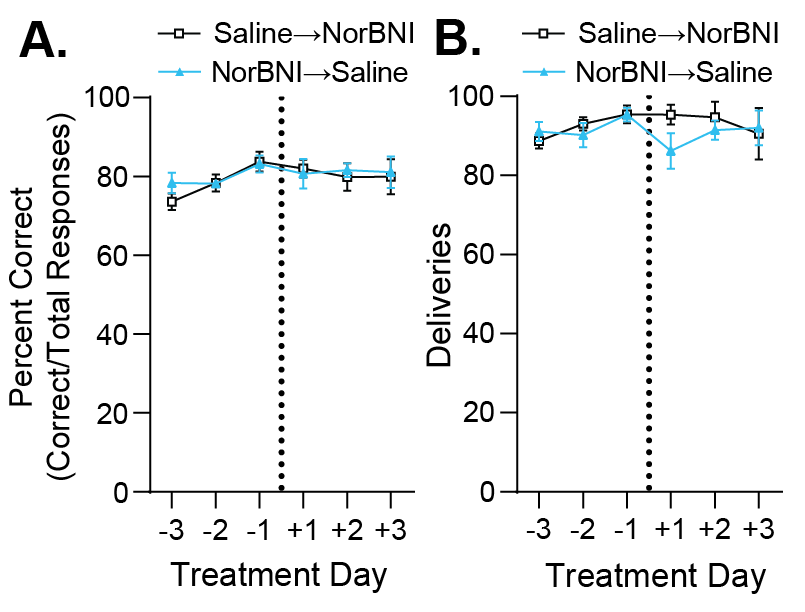


**Supplemental Figure 2. Systemic KOR blockade did not alter performance of a previously learned contingency during positive reinforcement.** Performance during positive reinforcement sessions 3 days before and 3 days after the crossover treatment. **(A)** The percent of responses that were correct (correct responses/total responses) per session before and after treatment for saline🡪NorBNI mice and NorBNI🡪saline mice. **(B)** The number of sucrose deliveries per session before and after treatment for saline🡪NorBNI mice and NorBNI🡪saline mice. Values indicate mean ± SEM. (n = 9, NorBNI🡪saline; n = 7, saline🡪NorBNI)


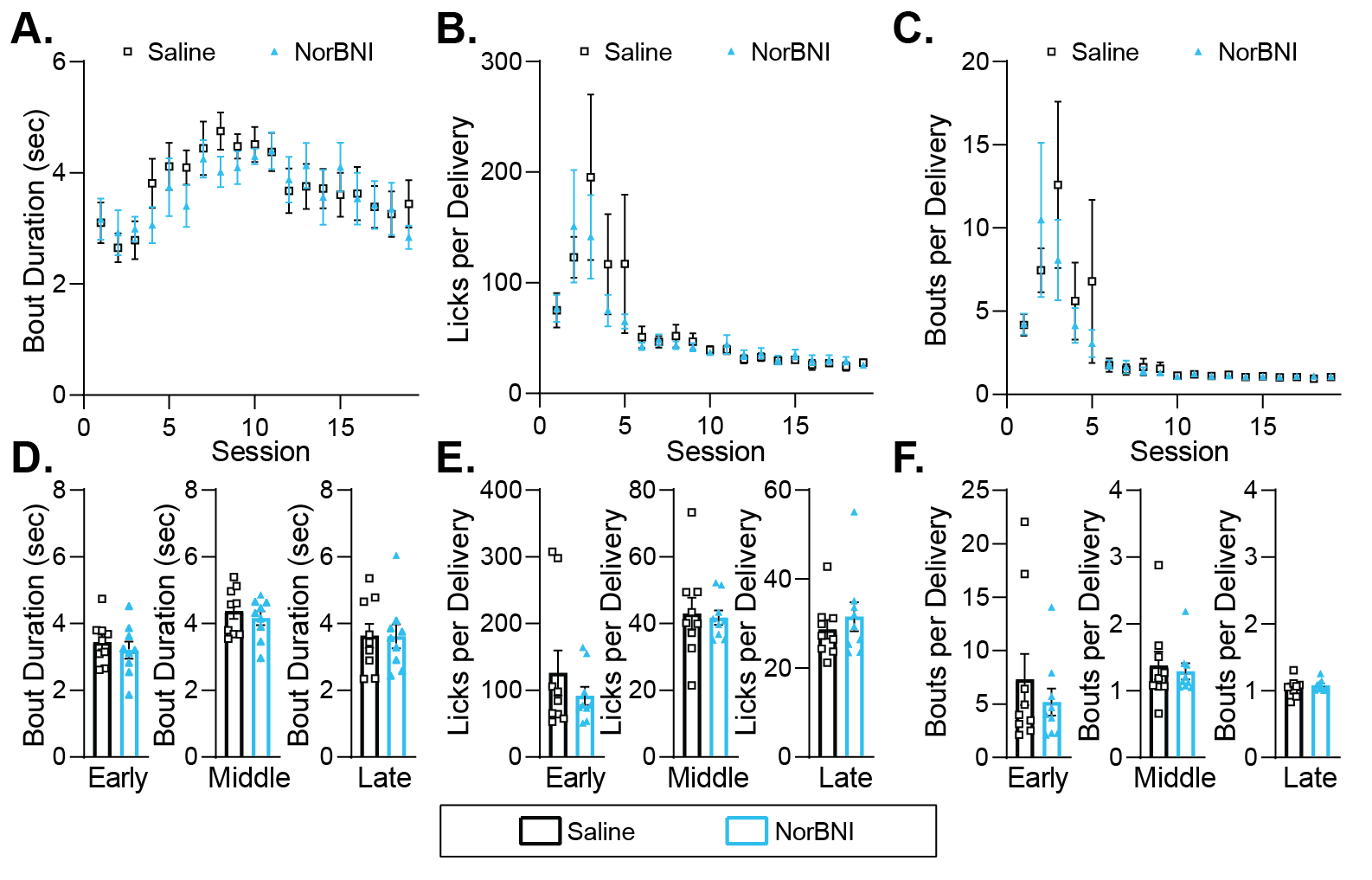


**Supplemental Figure 3. Systemic KOR antagonism had no effect on consummatory patterns during positive reinforcement learning. (A-C)** Lick microstructure analyzed across positive reinforcement sessions showed that NorBNI had no effect on consummatory behaviors over the course of learning. **(A)** There was no difference in the average bout duration between NorBNI and saline groups, though the bout length did change over sessions (two-way mixed-model ANOVA, session, F_(3.809, 57.56)_ = 4.869, p = 0.0022; treatment, F_(1, 16)_ = 0.2213, p = 0.6444; session x treatment, F_(18, 272)_ = 0.5977, p = 0.9002). **(B)** Similarly, average number of licks per sucrose delivery varied over sessions but was not altered by NorBNI treatment (two-way mixed-model ANOVA, session, F_(2.239, 33.58)_ = 7.553, p = 0.0014; treatment, F_(1, 16)_ = 0.3597, p = 0.5571; session x treatment, F_(18, 270)_ = 0.5117, p = 0.9518), **(C)** and the same was true for average number of bouts per delivery (two-way mixed-model ANOVA, session, F_(2.188, 32.70)_ = 7.530, p = 0.0016; treatment, F_(1, 16)_ = 0.2913, p = 0.5968; session x treatment, F_(18, 269)_ = 0.5837, p = 0.9101). **(D-F)** To ensure that potential timepoint specific effects were not overlooked, values were averaged into *Left:* early (sessions 1-6), *Center:* middle (sessions 7-12), and *Right:* late (sessions 13-19) epochs. **(D)** NorBNI had no effect on the average bout duration during early (unpaired t-test, t_16_ = 0.7111, p = 0.4873), middle (unpaired t-test, t_16_ = 0.6949, p = 0.4971), or late (unpaired t-test, t_16_ = 0.02974, p = 0.9766) epochs. **(E)** There was no effect of KOR blockade on the average number of licks per sucrose delivery across early (unpaired t-test, t_16_ = 0.9395, p = 0.3614), middle (unpaired t-test, t_16_ = 0.2327, p = 0.8189), or late (unpaired t-test, t_16_ = 0.7314, p = 0.4751) epochs. **(F)** NorBNI treatment did not alter average number of bouts per sucrose delivery across early (unpaired t-test, t_16_ = 0.7806, p = 0.4464), middle (unpaired t-test, t_16_ = 0.3518, p = 0.7296), or late (unpaired t-test, t_16_ = 0.6360, p = 0.5337) epochs. Values indicate mean ± SEM. (n = 9 per group)


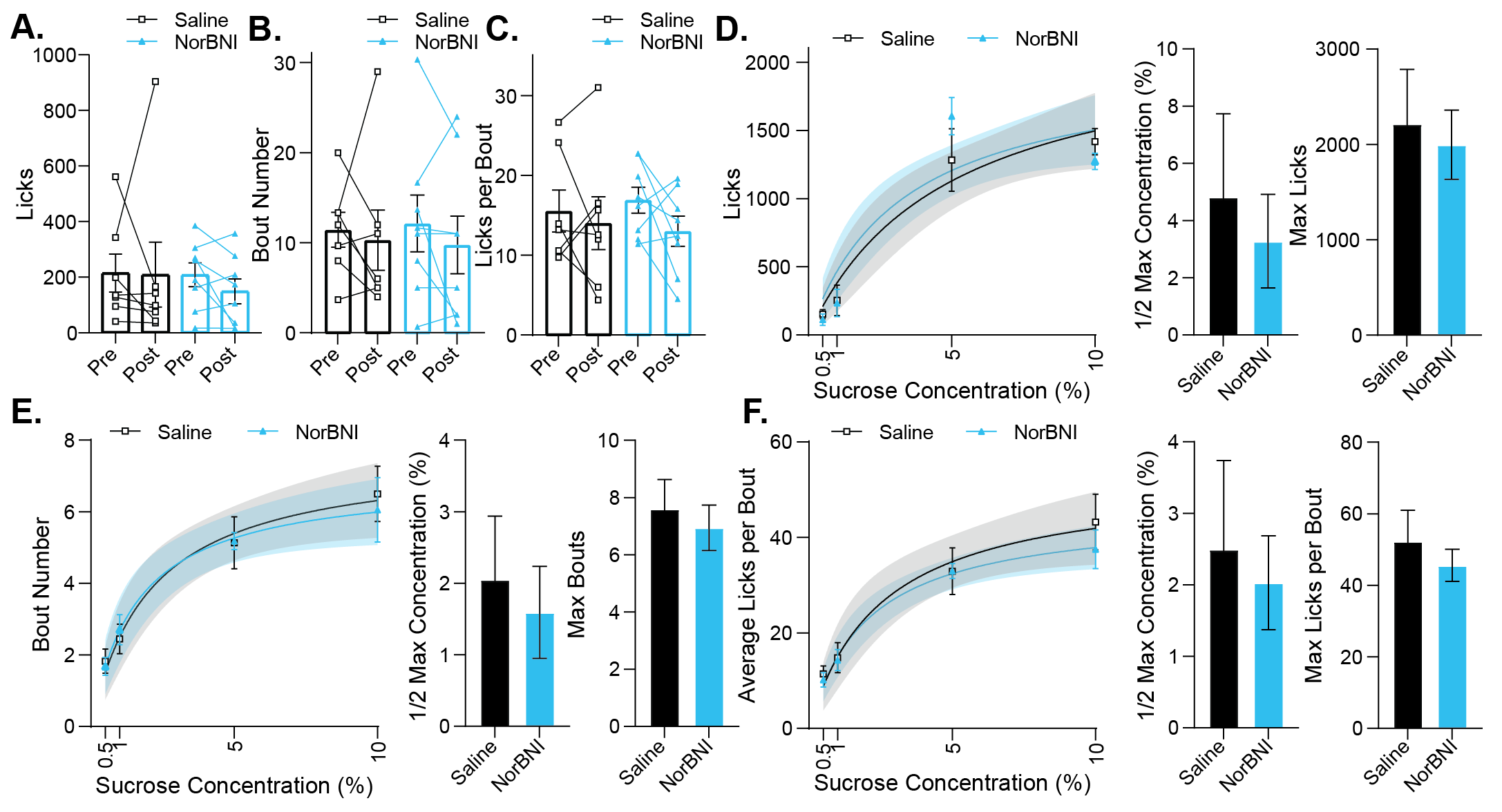
**Supplemental Figure 4. Increased rate of positive reinforcement learning was not due to a change in unconditioned response to sucrose.** A two-bottle choice design was used to determine whether NorBNI alters the response to sucrose independent of reinforcement learning. Mice ran three days of baseline with 1% (w/v) sucrose access, followed by treatment with NorBNI (10 mg/kg) or saline before one additional day of testing with 1% sucrose access for within-subject comparison. Next, a dose response curve of sucrose concentrations was generated over days for between subject comparison. **(A-C)** Within-subject comparison of lick microstructure during consumption of 1% (w/v) sucrose before and after NorBNI (or saline) treatment. **(A)** There was no difference in total licks per session between the baseline and post-treatment days or due to treatment with NorBNI (two-way mixed-model ANOVA, session, F_(1, 13)_ = 0.3566, p = 0.5606; treatment, F_(1, 13)_ = 0.1560, p = 0.6993). Neither NorBNI nor saline treatment resulted in a change in the number of licks for 1% sucrose (planned within-subject Šidák multiple comparisons test, pre- vs. post- treatment; saline, t_13_ = 0.06970, p = 0.9970; NorBNI, t_13_ = 0.7997, p = 0.6844). **(B)** NorBNI and saline groups showed no difference in the number of bouts for 1% sucrose (two-way mixed-model ANOVA, session, F_(1, 13)_ = 0.8555, p = 0.3719; treatment, F_(1, 13)_ = 0.0004467, p = 0.9835). There was no difference in the number of bouts for either group in a pre- vs. post- treatment comparison of microstructure (planned within-subject Šidák multiple comparisons test, pre- vs. post- treatment; saline, t_13_ = 0.4115, p = 0.9023; NorBNI, t_13_ = 0.9141, p = 0.6123). **(C)** There was no difference in the average number of licks per bout between groups for 1% sucrose before and after treatment (two-way mixed-model ANOVA, session, F_(1, 13)_ = 2.152, p = 0.1662; treatment, F_(1, 13)_ = 0.004255, p = 0.9490). Neither NorBNI nor saline treatment resulted in a change in the number of licks per bout for 1% sucrose (planned within-subject Šidák multiple comparisons test, pre- vs. post- treatment; saline, t_13_ = 0.5629, p = 0.8262; NorBNI, t_13_ = 1.546, p = 0.2710). **(D-F)** *Left:* Dose response best-fit nonlinear curves for lick microstructure with 95% confidence interval bands and *Center:* a comparison of the curve half-max sucrose concentration and *Right:* the curve maximum lick behavior. **(D)** There was no difference between NorBNI and saline groups in the half-maximal concentration (unpaired t-test, t_13_ = 0.4787, p = 0.6401) or curve-fit maximum (unpaired t-test, t_13_ = 0.3366, p = 0.7418) for the number of licks across sucrose concentrations. **(E)** After treatment, there was no difference in the half-maximal concentration (unpaired t-test, t_13_ = 0.4251, p = 0.6777) or maximum (unpaired t-test, t_13_ = 0.5169, p = 0.6139) for the number of bouts across concentrations between NorBNI and control groups. **(F)** Across sucrose concentrations, there was also no difference in the half-maximal concentration (unpaired t-test, t_13_ = 0.3482, p = 0.7333) or curve maximum for average licks per bout (unpaired t-test, t_13_ = 0.7281, p = 0.4794). Values indicate mean ± SEM unless otherwise noted. (NorBNI, n = 8; saline, n = 7)


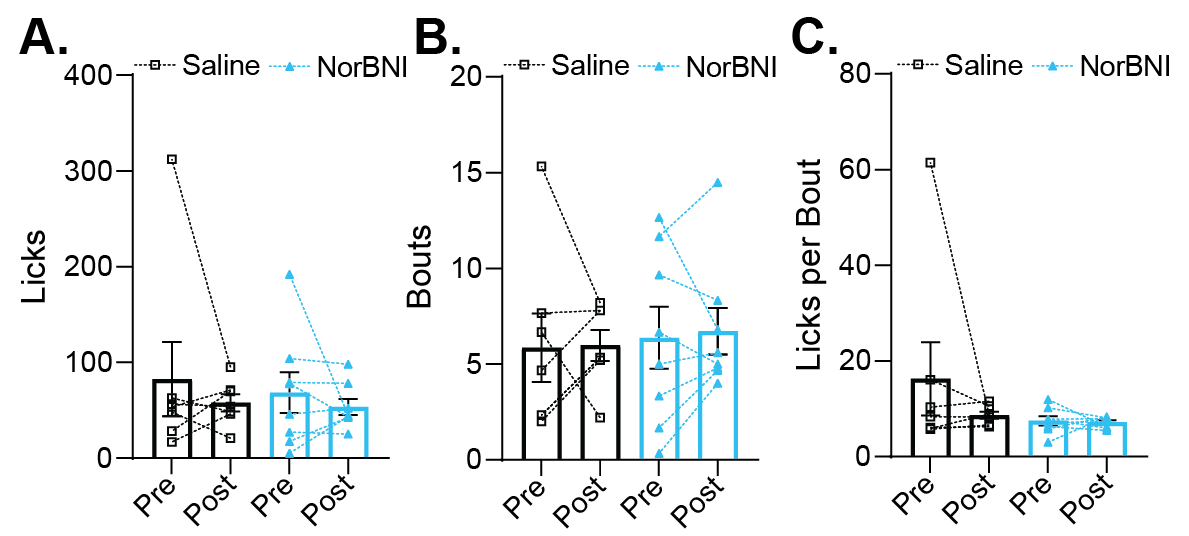


**Supplemental Figure 5.** **NorBNI did not alter water consumption during two-bottle choice sessions. (A)** There was no effect on the number of licks for water before or after treatment (two-way mixed-model ANOVA, session, F_(1, 13)_ = 1.092, p = 0.3151; treatment, F_(1, 13)_ = 0.1358, p = 0.7184) and neither treatment with NorBNI nor saline had an effect on licks for water when comparing pre- to post- treatment (planned within-subject Šidák multiple comparisons test, pre- vs. post- treatment; saline, t_13_ = 0.8878, p = 0.6289; NorBNI, t_13_ = 0.5807, p = 0.8163). **(B)** There was no difference in the number of bouts as an effect of treatment or session (two-way mixed-model ANOVA, session, F_(1, 13)_ = 0.05743, p = 0.8143; treatment, F_(1, 13)_ = 0.1249, p = 0.7294) and treatment with neither NorBNI or saline had an effect on the number of bouts for water when comparing pre- to post- treatment (planned within-subject Šidák multiple comparisons test, pre- vs. post- treatment; saline, t_13_ = 0.08807, p = 0.9953; NorBNI, t_13_ = 0.2566, p = 0.9606). **(C)** There was no effect of treatment or session on the average number of licks per bout for water (two-way mixed-model ANOVA, session, F_(1, 13)_ = 1.198, p = 0.2935; treatment, F_(1, 13)_ = 1.966, p = 0.1843) and neither treatment with NorBNI nor saline had an effect on licks per bout for water when comparing pre- to post- treatment (planned within-subject Šidák multiple comparisons test, pre- vs. post- treatment; saline, t_13_ = 1.447, p = 0.3137; NorBNI, t_13_ = 0.05563, p = 0.9981). Values indicate mean ± SEM. (NorBNI, n = 8; saline, n = 7)


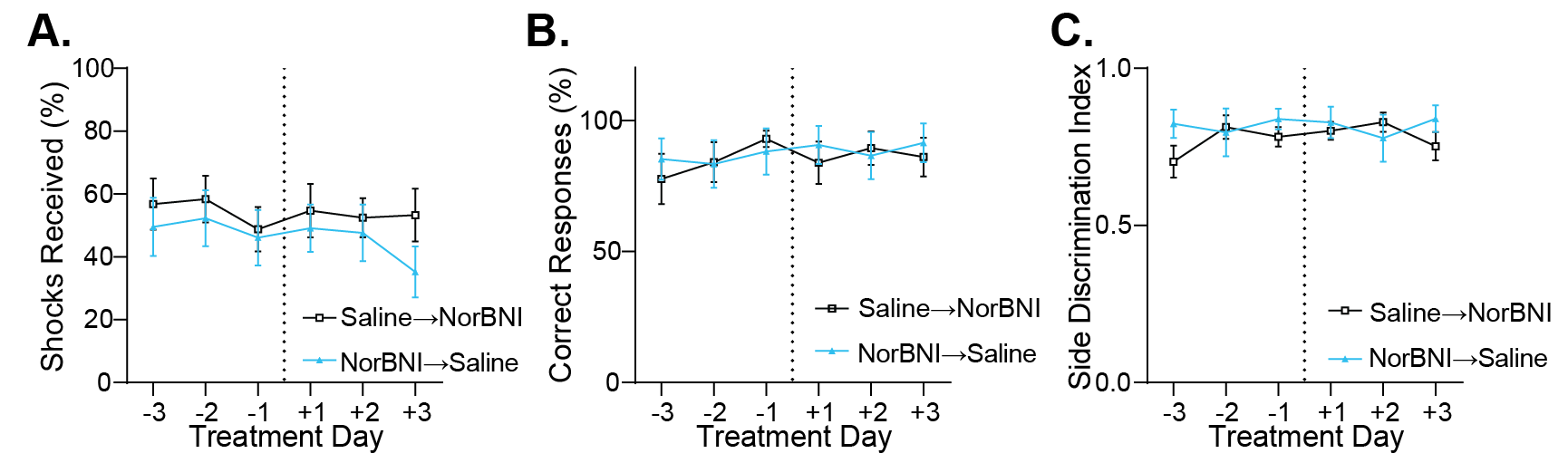


**Supplemental Figure 6.** **Systemic KOR blockade did not alter performance of a previously learned contingency during negative reinforcement.** Raw values of performance during negative reinforcement sessions 3 days before and 3 days after crossover treatment. **(A)** The percentage of possible shocks received per session ([shocks received/220 shocks possible]x100) before and after treatment for saline🡪NorBNI mice and NorBNI🡪saline mice. **(B)** The percentage of S^D^ presentations during which a correct response was made ([correct responses/S^D^ presentations]x100) per session before and after treatment for saline🡪NorBNI mice and NorBNI🡪saline mice. **(C)** Side discrimination index demonstrated per session before and after treatment for saline🡪NorBNI mice and NorBNI🡪saline mice. Values indicate mean ± SEM. (NorBNI, n = 9; saline, n = 10)

**
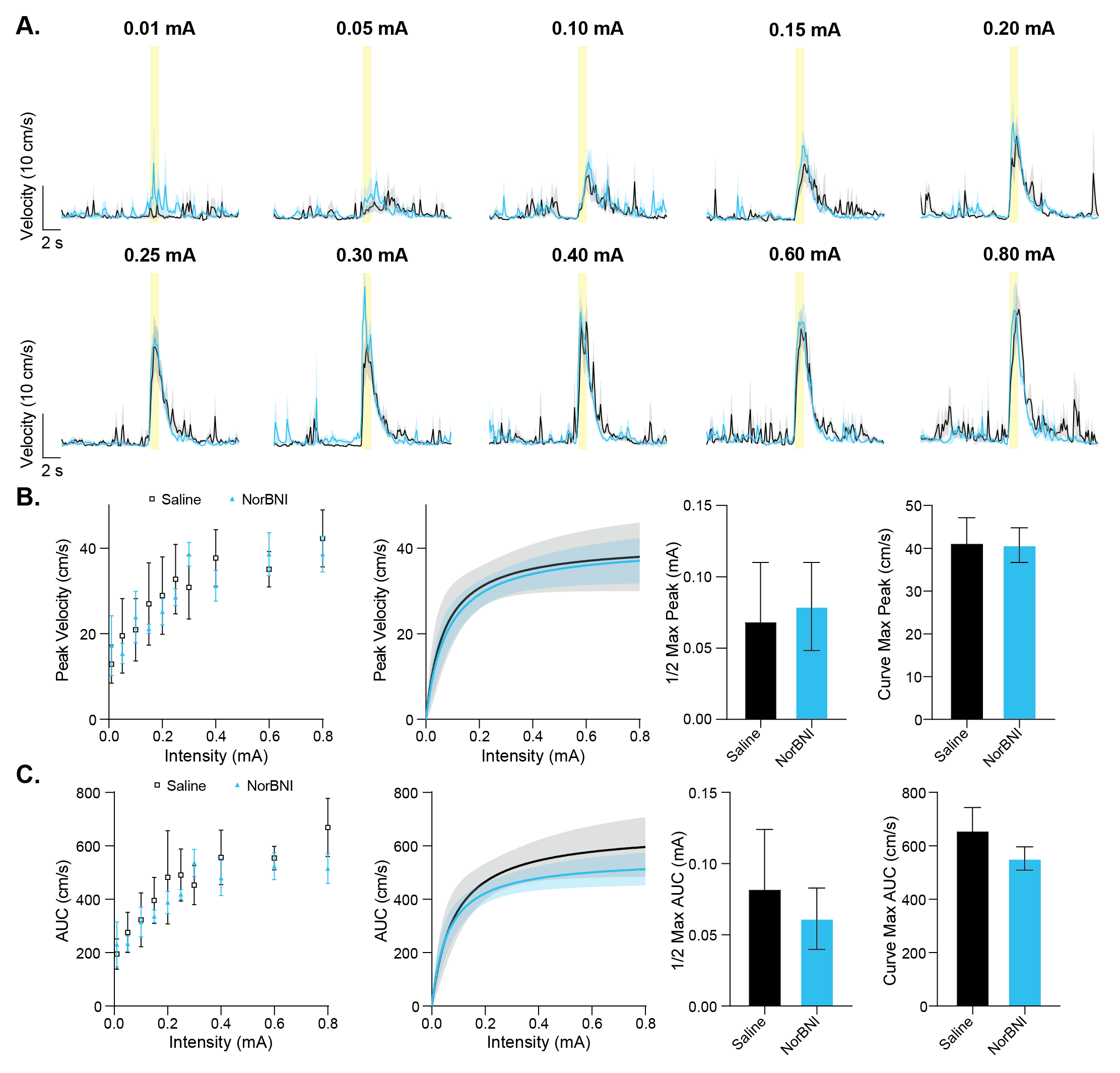
Supplemental Figure 7. KOR antagonism did not alter unconditioned response to footshock. (A)** Raw traces of mouse velocity over time, aligned around footshock delivery (stimulus onset and offset indicated by yellow shading). **(B-C)** *Left:* Group averages of measures of response were plotted across footshock intensities. *Center:* The best nonlinear fit with 95% confidence interval bands is shown. *Right:* Half-maximal intensity and curve maximums were calculated and compared via a t-test. **(B)** KOR blockade had no effect on the peak velocity within 0.5 seconds before, and 5 seconds after, each shock onset. When curve fit, there was no difference in the half-maximal shock amplitude (unpaired t-test, t_8_ = 0.2022, p = 0.8448) or the curve maximum peak velocity (unpaired t-test, t_8_ = 0.07231, p = 0.9441) between groups. **(C)** The area under the curve was taken from 0.5 seconds prior to, and 5 seconds after, the onset of the footshock. There was no difference in the half-maximal intensity (unpaired t-test, t_8_ = 0.4442, p = 0.6687) or maximum velocity (unpaired t-test, t_8_ = 1.088, p = 0.3081) as taken from the best curve fit. Values indicate mean ± SEM unless otherwise noted. (n = 5 per group)


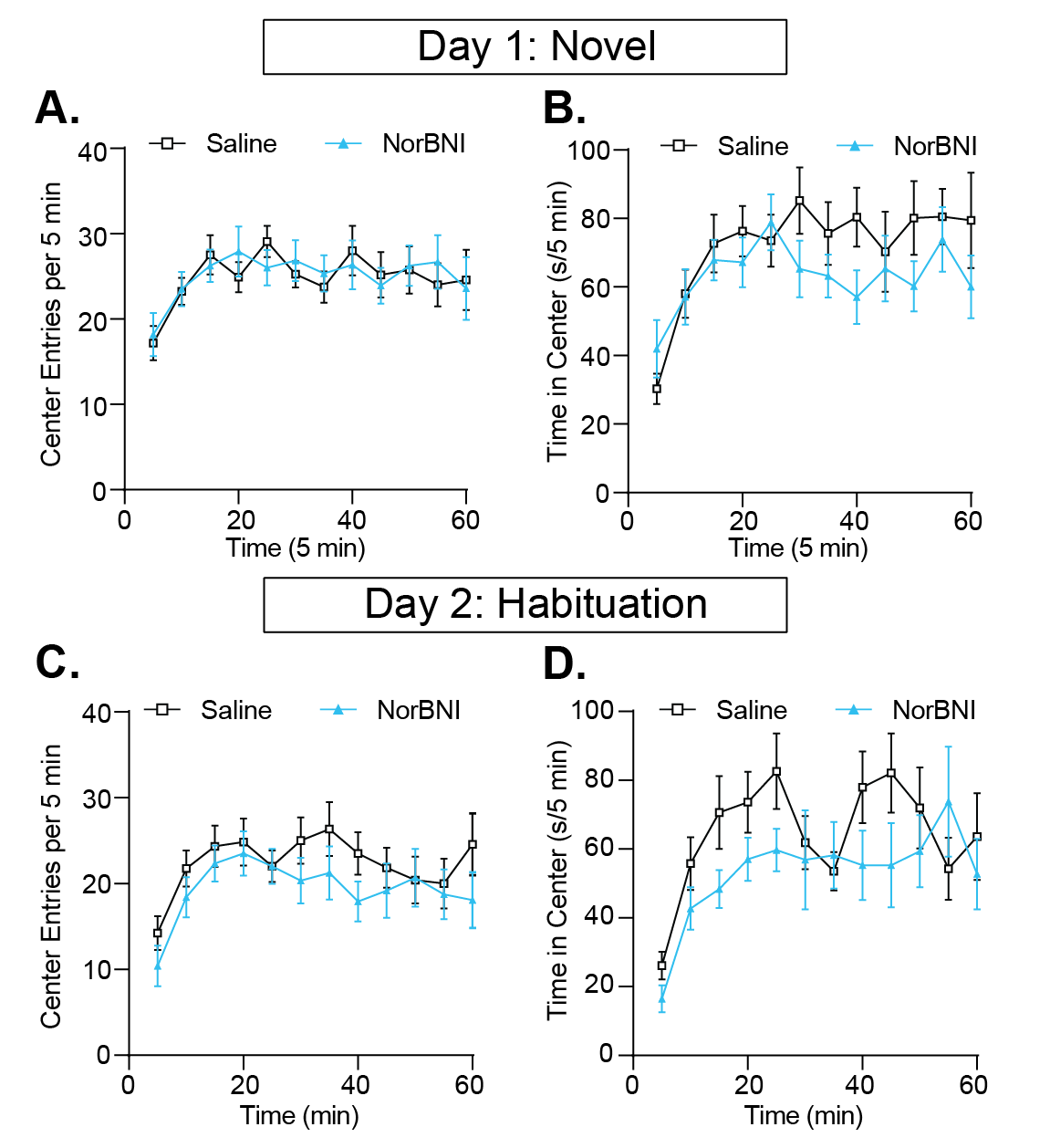


**Supplemental Figure 8. KOR blockade did not alter center time in an open field.** Entries and time spent in the center of the open field arena during novelty and habituation sessions. **(A)** Both groups showed the expected increase in frequency of entries into the center over the course of the session, but there was no effect of treatment (two-way ANOVA, time, F_(5.786, 127.3)_ = 4.629, p = 0.0003; treatment, F_(1, 22)_ = 0.005713, p = 0.9404; time x treatment, F_(11, 242)_ = 0.5805, p = 0.8440). **(B)** Similarly, mice spent more time in the center of the area over the course of the session, but center time was not altered by NorBNI treatment (two-way ANOVA, time, F_(6.354, 139.8)_ = 6.087, p < 0.0001; treatment, F_(1, 22)_ = 0.9989, p = 0.3284; time x treatment, F_(11, 242)_ = 1.460, p = 0.1473). **(C)** During the second session, there was an increase in entries into the center of the arena, but no effect of KOR antagonism (two-way ANOVA, time, F_(4.848, 106.7)_ = 5.971, p < 0.0001; treatment, F_(1, 22)_ = 1.071, p = 0.3121; time x treatment, F_(11, 242)_ = 0.7502, p = 0.6894). **(D)** There was no effect of NorBNI on the cumulative duration spent in the center during the second session as compared to saline, though both groups increased time spent in the center over time (two-way ANOVA, time, F_(4.762, 104.8)_ = 5.703, p = 0.0001; treatment, F_(1, 22)_ = 1.713, p = 0.2041; time x treatment, F_(11, 242)_ = 1.403, p = 0.1721). Together these data demonstrate that NorBNI treatment did not impact avoidance of the center of the area, which is thought to be a readout of anxiogenesis, or the rate at which avoidance of the center dissipated over the course of experience. Entries and time spent in the center area both displayed clear novelty-dependent expression, whereby avoidance behavior habituated as a function of novelty, confirming that our measurement was sensitive to neophobic avoidance which occurs in addition to novelty-independent avoidance driven by thigmotaxis and anxiety-like behaviors. Values indicate mean ± SEM. (n = 12 per group)


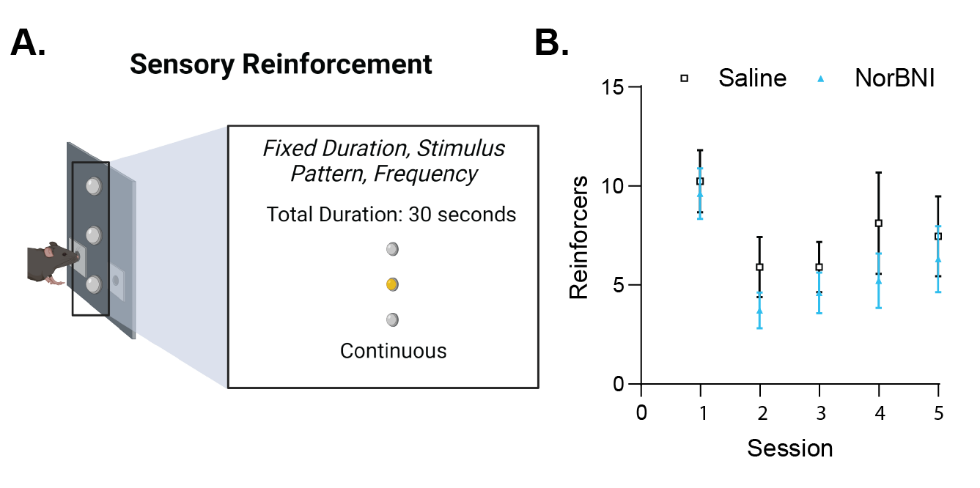


**Supplemental Figure 9. Blockade of KORs had no effect on responding for a non-novel visual stimulus. (A)** Schematic of the task design during which an active response results in the illumination of a single cue light for 30 seconds. **(B)** There was no difference by treatment in responding for the illumination of a cue light for 30 seconds (two-way ANOVA, time, F_(2.409, 40.96)_ = 5.077, p = 0.0073; treatment, F_(1, 17)_ = 1.147, p = 0.2992; time x treatment, F_(4, 68)_ = 0.2620, p = 0.9013). Values indicate mean ± SEM. (NorBNI, n = 10; saline, n = 9)

1. Robinson, S. L. & McCool, B. A. Microstructural analysis of rat ethanol and water drinking patterns using a modified operant self-administration model. *Physiol Behav* **149**, 119–30 (2015).

2. Spector, A. C., Klumpp, P. A. & Kaplan, J. M. Analytical issues in the evaluation of food deprivation and sucrose concentration effects on the microstructure of licking behavior in the rat. *Behavioral neuroscience* **112**, 678–94 (1998).

3. Naneix, F., Peters, K. Z. & McCutcheon, J. E. Investigating the Effect of Physiological Need States on Palatability and Motivation Using Microstructural Analysis of Licking. *Neuroscience* **447**, 155–166 (2020).

4. Kelley, A. E. *Locomotor Activity and Exploration*. *Handbook of Behavioral Neuroscience* vol. 10 (Elsevier B.V., 1993).

5. Montiglio, P.-O., Garant, D., Thomas, D. & Réale, D. Individual variation in temporal activity patterns in open-field tests. *Anim Behav* **80**, 905–912 (2010).

6. Weiss, I. C., Pryce, C. R., Jongen-Rêlo, A. L., Nanz-Bahr, N. I. & Feldon, J. Effect of social isolation on stress-related behavioural and neuroendocrine state in the rat. *Behavioural Brain Research* **152**, 279–295 (2004).

7. Walsh, R. N. & Cummins, R. A. The open-field test: A critical review. *Psychol Bull* **83**, 482–504 (1976).

8. Clinton, S. M. *et al.* Individual differences in novelty-seeking and emotional reactivity correlate with variation in maternal behavior. *Horm Behav* **51**, 655–664 (2007).

9. Kabbaj, M., Devine, D. P., Savage, V. R. & Akil, H. Neurobiological Correlates of Individual Differences in Novelty-Seeking Behavior in the Rat: Differential Expression of Stress-Related Molecules. *The Journal of Neuroscience* **20**, 6983–6988 (2000).

10. Leach, A. C., Pitts, E. G., Siciliano, C. A. & Ferris, M. J. α7 nicotinic acetylcholine receptor modulation of accumbal dopamine release covaries with novelty seeking. *European Journal of Neuroscience* **55**, 1162–1173 (2022).
